# The implementation of embedded researchers in policy, public services, and commercial settings: a systematic evidence and gap map

**DOI:** 10.1186/s43058-024-00570-3

**Published:** 2024-04-16

**Authors:** Dylan Kneale, Claire Stansfield, Rebecca Goldman, Sarah Lester, Rachael C. Edwards, James Thomas

**Affiliations:** 1 EPPI Centre, UCL Social Research Institute, University College London, London, UK; 2 Independent Consultant, London, UK

**Keywords:** Systematic map, Embedded researchers, Knowledge mobilisation, Knowledge translation, Research use, Evidence use, Evidence-informed decision-making, Research activity

## Abstract

**Background:**

Embedding researchers into policy and other settings may enhance research capacity within organisations to enable them to become more research active. We aimed to generate an evidence map on evaluations of embedded researcher interventions to (i) identify where systematic reviews and primary research are needed and (ii) develop conceptual understandings of ‘embedded researchers’. We define ‘embedded researchers’ through a set of principles that incorporate elements such as the aim of activities, the types of relationships and learning involved, and the affiliations and identities adopted.

**Methods:**

We included studies published across all sectors, searching fourteen databases, other web sources and two journals for evaluations published between 1991 and spring 2021. Data were extracted using a coding tool developed for this study. We identified new typologies of embedded researcher interventions through undertaking Latent Class Analysis.

**Results:**

The map describes 229 evaluations spanning a variety of contexts. Our set of principles allowed us to move beyond a narrow focus on embedded researchers in name alone, towards consideration of the wide range of roles, activities, identities, and affiliations related to embedded researchers. We identified 108 different allied terms describing an embedded researcher. Embedded researcher activity spanned a continuum across lines of physical, cultural, institutional, and procedural embeddedness (from weaker to more intense forms of embeddedness) and took a range of forms that bridge or blur boundaries between academia and policy/practice.

**Conclusions:**

We developed a broad map of international embedded researcher activity in a wide range of sectors. The map suggests that embedded researcher interventions occupy a broader suite of models than previously acknowledged and our findings also offer insight on the type and nature of this literature. Given the clear policy interest in this area, a better understanding of the processes involved with becoming embedded within an organisation is needed. Further work is also necessary to address the challenges of evaluating the work of embedded researchers, including consideration for which outcome measures are most appropriate, to better understand their influence.

**Supplementary Information:**

The online version contains supplementary material available at 10.1186/s43058-024-00570-3.

Contributions to the literature

- Embedded researchers are a model of working that can bridge the gap between cultures of research generation and research use.
- Narrow definitions of embedded researchers used within previous studies have focussed on academics being physically situated within policy/practice settings, but this can mean that models sharing similar characteristics and aims are overlooked.
- This systematic map of the literature identifies several forms of embedded research and adopts novel methods to identify typologies of these models.
- The map demonstrates that embedded researcher interventions take different forms and embedded researchers adopt different strategies and undertake a variety of activities in helping organisations become research active.

## Background

### Introduction

Collaborative approaches between researchers and research users, including knowledge transfer partnerships and embedded researchers, are gaining traction as promising ways to bridge the gap between research and practice [1, 2]. While dependent on a number of factors to succeed [3, 4], embedded researchers, with their dual affiliation, have been hypothesised to act as catalysts for change in developing research cultures and capacity through shared, mutually beneficial learning processes.

### What is an embedded researcher?

Embedded researchers have previously been defined as those ‘researchers who work inside host organisations as members of staff, while also maintaining an affiliation with an academic institution’ [5]. McGinity and Salokangas [6] define embedded researchers as “individuals or teams who are either university-based or employed undertaking explicit research roles within […] other organizations with the purpose of identifying and implementing a collaborative research agenda.” Both definitions are ambiguous in terms of the activities which embedded researchers undertake and whether they encompass research-based activities such as facilitating research, advising research production (e.g. providing advice on funding opportunities, research methods, or ethics), or mediating and interpreting research. Other interpretations implicitly suggest that embedded researcher activities necessitate conducting research and that ‘embedded researchers carry out research alongside the end users, as part of that context’ [1, 6], we view the remit of an embedded researcher as broader than solely involving the generation of research in situ. Rather, their remit may include taking steps towards catalysing longer-term organisational research active cultures.

Other noteworthy features of the definitions proposed above are (i) an emphasis on dual affiliation and (ii) ambiguity on the direction of embeddedness. Regarding dual affiliation, in some models of placement, embedded researchers may not feel, or may not be recognised as having dual affiliation or team membership and we may consider that different models have different degrees and types of embeddedness. Marshall and colleagues [7] recognise that embedded researchers can experience tensions between immersion and distance, and challenge and support, whilst acting as a “critical friend” and “fresh pair of eyes”. Similarly, most definitions suggest that embedded researcher models are unidirectional, involving academic researchers being embedded into policy or practice organisations. This excludes models involving representatives from policy or practice organisations being embedded to undertake research activities within academic or other research settings. A unidirectional focus also speaks to maintaining a hierarchy in research and evidence production systems, where only academic researchers are perceived as having the skills to create change within policy/practice settings and to create a collaborative research agenda. Furthermore, such interpretations also serve to overlook pre-existing research skills and practices within policy/practice settings, which an embedded researcher may enhance rather than create anew. This is particularly problematic for some settings such as public health, where the workforce tends to have strong pre-existing research skills but where teams are not necessarily engaging in research activities to their full potential.

Rather than fitting within a crisp, neat definition, we contest that embedded researcher activity may instead be more usefully conceptualised through a set of principles. Previous evidence syntheses suggest that embedded researcher activities take place using a variety of allied terms (e.g. knowledge broker or insider researcher) [8], which may be unified through a common set of principles or ‘intervention components’. The following principles were developed with the aid of an Advisory Group for the project and operationalised in our systematic map to identify embedded researchers according to:

i. Purpose and activities: they enable research activity and research use. For example, they may undertake research, facilitate the conduct of research (through sourcing data, creating data sharing arrangements or advising or training on research/policy processes), and support research use. Through these actions they have the potential to enhance cultures of research activity.
ii. Dual affiliation: they are *co-located* — but not necessarily physically — in a defined policy, practice or commercial formal organisation and they have an affiliation with an academic institution or research organisation, and/or their post is specifically funded by an academic institution or research organisation.
iii. Setting: they are situated within a host team (physically, institutionally, and/or culturally) and/or are expected to work within the host team culture for a high proportion of their time as a team member working on and applying research to solve practical problems or building research capacity (this latter characteristic is shared with definitions put forward around researchers-in-residence, an allied term [9]).
iv. Transformative ways of working: embedded researcher activities entail continued engagement with a host team (i.e., an embedded researcher is more than a notional job title but a different way of working for researchers and practitioners who are embedded).
v. Relational and time-limited: the relational nature of embedded research necessitates that this is a longer-term activity relative to other ways of enhancing research cultures (e.g. providing short-term training sessions). However, embedded researcher activities are also time limited owing to their role in changing cultures, and embedded researchers are not permanent members of staff within their host organisation.
vi. Organisational-level oversight: we expect that host organisations will be able to influence and direct the work of embedded researchers (i.e., embedded researcher activities are often distinct from, for example, an ethnographic study of policy-making in an organisation).
vii. Experienced professionals: we view embedded researchers as experienced professionals who contribute to and build upon pre-existing skills and experience within the host organisation. Therefore, we do not view taught degree placements for undergraduate dissertations and research projects as examples of embedded researcher activity; doctoral research is included if other conditions are met, for example the policy/practice organisation can influence the work as in condition vi.
viii. Two-way organisational learning: embedded researchers exemplify a two-way relationship where there is learning to be gained for both organisations. Our definition also leaves open the possibility of bi-directionality in that researchers could be embedded into policy/practice settings and that those from policy/practice settings could be embedded into research organisations (provided they meet the other principles).

For the purposes of creating the map, we also view embedded researchers as organisational-level interventions, aiming (explicitly or implicitly) to create change in research cultures within organisations (hosting and/or sending organisations). This means we do not include within our scope researchers who are embedded within communities where there is no defined organisation.

### Objectives of the systematic map

The objectives of this map are:

- To generate a systematic map of evidence on evaluations of embedded researchers in policy, public services, commercial or industry settings (public, private and third sectors).
- To develop conceptual understandings of what the term ‘embedded researcher’ means.
- To identify areas where systematic reviews of embedded researcher evaluations are needed (and feasible).
- To identify gaps in evidence where further primary research is needed.

This map is intended to build on previous work that has synthesised evidence on embedded researchers [for example 1, 5, 8] through (i) having a more inclusive focus on embedded researcher models including those which may be known under a different name (e.g. researcher-in-residence, industrial doctorate, participatory action research); (ii) conceptualising 'embeddedness' broadly by including instances where there was no substantial physical co-location; (iii) drawing on evidence across sectors and drawing on evidence beyond the UK; (iv) identifying typologies of embedded researcher models; (v) expanding on the concepts incorporated in the search strategy; and (vi) presenting the results visually to communicate the evidence through EPPI-Mapper and EPPI-Visualiser [10].

## Methods

### Protocol

The protocol for this map was published on the EPPI Centre website [11] and on the Open Science Framework [12].

### Search

The search strategy, designed by an Information Scientist, aimed to be comprehensive in identifying embedded researcher evaluations within the field of healthcare and public health. It also aimed to be expansive in capturing a wide range of literature from other scientific disciplines that inform policy, practice, or industrial sectors across different geographical contexts. This included searching 14 databases, the contents of two journals (Evidence and Policy and Research for All) and browsing 15 websites.

The following bibliographic databases were searched between 19 May and 3 June 2021 to identify research published in English since 1991:

- Health research databases: CINAHL (EBSCO), MEDLINE (OVID) PsycINFO (OVID)
- Social science and Social Policy databases: ASSIA (Proquest), Book Citation Indexes – Science/Social Science and Humanities (Web of Science), Emerging Sources Citation Index (Web of Science), Health Management Information Consortium (OVID), International Bibliography of Social Sciences (Proquest), Social Policy and Practice (OVID), Social Science Citation Index (Web of Science), Sociological Abstracts (Proquest)
- Business/ Education/Science databases: ABI inform (Proquest), Business Source Premier (EBSCO), Econlit (EBSCO), Educational Administration Abstracts (EBSCO), Educational Research Information Center (EBSCO), Science Citation Index (Web of Science)

Search terms were developed to reflect the following concepts and searched using free-text and controlled vocabulary where available.

1. Context: policy and practice interfaces, knowledge exchange, research engagement, healthcare settings
2. Interventions that seek to enable organisations to become more research active involving placement of academic researchers: embedded researcher, co-locating research, secondment, researcher-in-residence, boundary spanner, academic-industry partnerships, capacity-building, mentoring
3. Study design: quantitative, qualitative, and mixed-methods evaluation studies that measure implementation processes or outcomes as well as descriptive case studies and reflective studies.

A full strategy is available on request and an example strategy using Web of Science is reproduced in the supplementary materials, along with details of how the search was designed, and the non-database searches (Appendix 1).

### Inclusion criteria

Studies were included on the following basis (see Supplementary Materials, Appendix 2, for more fine-grained exclusion and inclusion criteria):

- Full-text paper published in English.
- Contains novel empirical data and/or detailed descriptions of the process of embedding researchers.
- Embedded researchers’ activity is focussed on enabling research or research use or research capacity within policy, practice, or commercial organisations.
- The embedded researcher is situated within the host team of a defined organisation (physically, institutionally, by affiliation, or culturally).
- The embedded researcher has an affiliation or receives funding from a research organisation.

We excluded studies where:

- The activities or influences of embedded researchers could not be disaggregated from larger projects or programmes (we termed these ‘meta-studies’).
- The researcher was embedded as part of a taught degree placement.
- The researcher was a permanent member of staff or on an open-ended placement.
- The host organisation could not influence the research/activities of the researcher or where there was no engagement with the host team.

### Identification of evidence: study selection

Results from literature searches were imported into to EPPI-Reviewer [10] and duplicates removed. Reviewers examined title and abstracts records for relevance and those relevant were screened at full text. Screening was undertaken initially in duplicate and then independently, having first piloted the inclusion and exclusion criteria to ensure consistency of screening decisions. The screening was supported by machine learning, and further details of the manual screening and machine learning elements are included in the supplementary materials (Appendix 3). Machine learning involved iteratively training a machine learning model to rank records by potential relevance, using the reviewers’ title and abstract screening decisions throughout the screening stage. This helped the team identify included studies earlier, and enabled screening to stop at an appropriate point. Machine learning was particularly advantageous in constructing this map as the range of terms through which embedded researchers are known meant that the search identified many irrelevant records, and manual screening was particularly slow owing to the close reading required to understand the abstracts.

### Coding and quality assessment

Coding reflected the following elements of embedded researcher (ER) interventions:

- Nature of the embeddedness (see Table 1).
- Direction of the embeddedness.
- Evaluation design.
- Country.
- Main focus of the ER (activities).
- Policy or practice sector within which ER is aiming to generate research activity (e.g. public health or education).
- Scope of the ER scheme and whether the model was trialled across a single or multiple organisations.
- Terminology used to describe the ER.

**Table 1:** Type of embeddedness and example evidence that support coding decisions (see Appendix 9 for fuller descriptions)

Type of embeddedness	Evidence to support
Physical co-location (confirmed)	*For example, through details provided about share of time spent or resources provided to support physical co-location (e.g. a desk)*
Culturally/institutionally	*For example, through being described as a team member in host team; to being embedded in team culture; to being set up to be staff member of host institution (e.g. through holding a joint contract or contract funded by host org — i.e., institutional barriers removed)*
Procedurally	*For example, through references to attending staff meetings; references to embedded researcher conducting specific tasks or occupying specific roles (e.g. tutor role or support role); through regular emails, calls *etc
Physical co-location (probable)	*For example, where physical co-location inferred as optional*
Confirmed as embedded, nature not described	*Description vague — i.e. researcher described as embedding but processes unclear*
Embeddedness sought but not achieved	*Where embeddedness was sought but was not achieved*

No quality assessment criteria were adopted for inclusion within the map, and the studies were not quality assessed individually; this mirrors the practice of creating systematic maps described elsewhere (for example [13, 14]). While individual studies were not quality assessed, quality assurance processes were implemented and are described in the supplementary materials (Appendix 4).

### Study visualisation

We used EPPI-Visualiser [10] to further understand the features of the map by generating frequencies, cross-tabs, and matrices from the initial coding. The main features of the map are also described narratively.

### Study mapping synthesis

We undertook Latent Class Analysis (LCA) (a statistical method that has not previously been applied to a systematic map to our knowledge) to identify different forms of embedded researcher interventions. LCA was identified as being a useful method in helping to understand variation in the types of embedded researcher interventions that were included in the map based on multiple characteristics simultaneously. While we may have been able to describe the contents of the map using frequency data based on a single variable (through a frequency table) or two variables (through a cross-tabulation), LCA offered the advantage of being able to examine how multiple characteristics related to one another simultaneously.

Embedded researcher interventions were distinguished based on the following observed characteristics: (i) the profile of activities undertaken; (ii) the nature of the embeddedness; and (iii) the direction of embeddedness. Different categories within these variables were assigned a numeric value and LCA was used to identify latent (unobserved) typologies of embedded researcher activity that represent latent structures in the data. In identifying latent typologies (classes) of embedded researcher interventions, we obtained parameters that give the proportions of individual studies within each of the latent classes (latent class probabilities) and the distribution of indicator variables within these classes (conditional probabilities). Together, these can identify both the characteristics of the typology and the probability of group membership for individual studies (the latter helping to determine the size of the group).

The optimal number of classes is determined by evaluating both the fit of the model, the interpretability of the classes, and the size of the classes. We evaluated the fit of different Latent Class solutions using a range of measures. Firstly, we considered the scaled relative entropy, which represents the degree to which the latent classes in the solution are distinct. This measure is analogous to R-squared, being bounded between 0 and 1 with higher values indicative of better model fit [15]. Secondly, we examine the Akaike’s Information Criteria (AIC) and Bayesian Information Criteria (BIC), taking the point of inflection as AIC and BIC increases with the number of classes as an indicator of ideal class size. In addition to examining the interpretability of the classes, the final consideration when determining the ideal class structure was the number of individual studies in each class and we avoided very small classes (typically containing less than 20 studies). This part of the analysis was carried out using STATA [16].

## Results

**Fig. 1:**
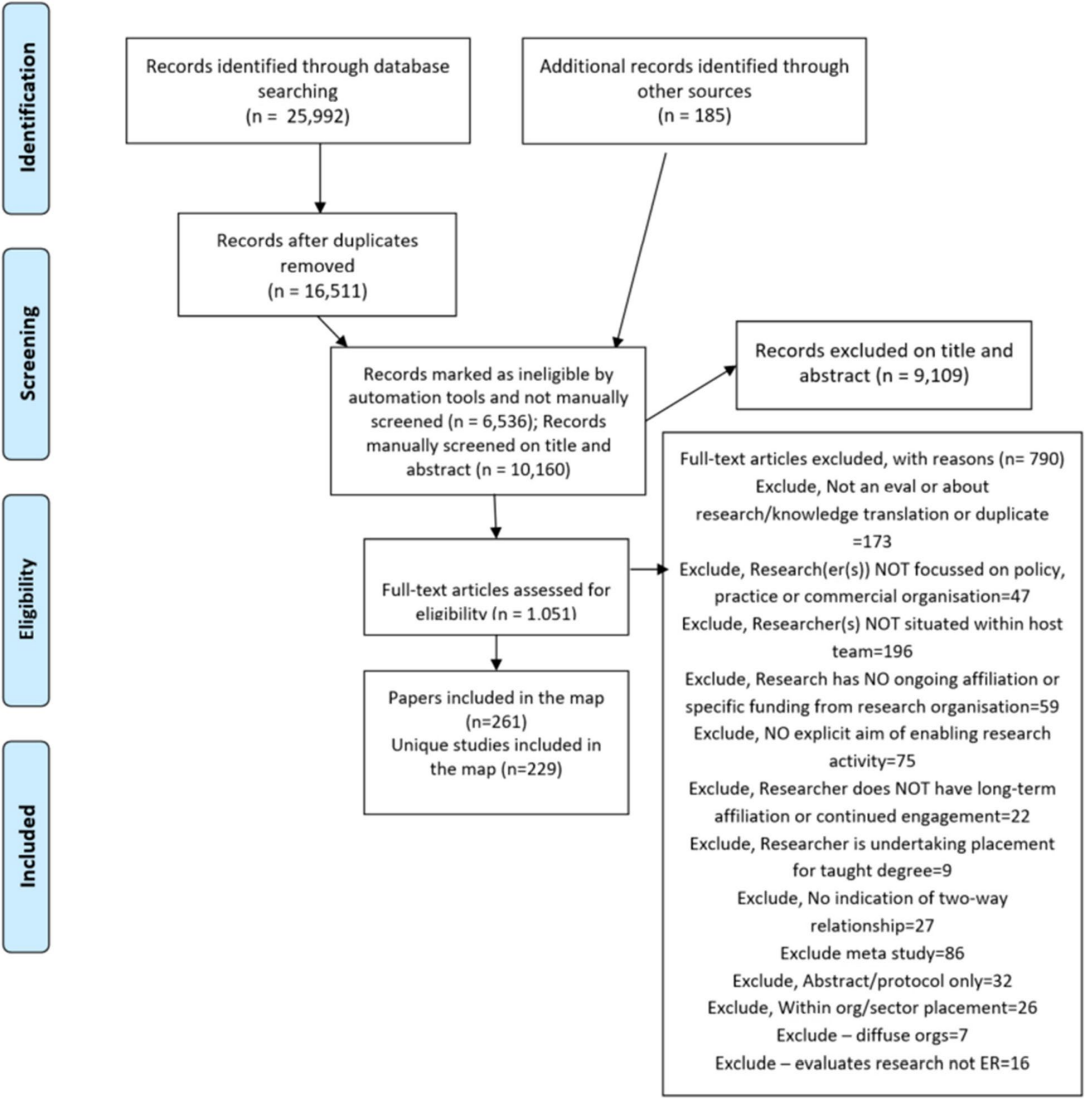
Flow of literature into the map

Out of the 16,747 citations identified, 10,171 were manually screened to identify 1058 potentially relevant citations based on title and abstract. After screening at full-text, 261 papers belonging to 229 studies were identified. The flow of studies into the map is shown in the PRISMA flowchart (Fig. 1), with the map including only unique studies (although a study may be supported by multiple papers). An interactive version of the map is available at https://eppi.ioe.ac.uk/eppi-vis/login/open?webdbid=60 through entering ‘60’ as the web database ID (if prompted) and selecting the button marked ‘View Map’; this resource allows users to construct different tables to help explore the features of the map that may be of interest. The supplementary materials also provide a list of included studies and linked papers (Appendix Table 5) as well as further details and full frequency plots for the dimensions of the map that have been coded. These are described narratively in the following sections (Appendix Tables 6–8).

### Study characteristics

The studies included in the map are from 1994 to 2021, primarily from the UK (*n* = 89 studies, 38.8%), USA (*n* = 53), Australia (*n* = 28), and Canada (*n* = 21) with activities conducted in a total of 47 countries or territories. More than half of the map comprises evaluations of embedded researcher activity taking place in health settings, including clinical health settings (primary or secondary care settings, *n* = 89, 38.8%) and public health settings (*n* = 35), with a smaller number evaluating activity in allied health settings (e.g. physiotherapy). In total, 33 sectors were represented where embedded researchers had been implemented. Other sectors included various forms of commercial or manufacturing industry (*n* = 33), education (*n* = 17), social care (*n* = 16), and crime and law enforcement (*n* = 10).

The studies followed two evaluation approaches: research involving structured tools, methods, or defined processes (*n* = 124), and those *only* adopting a reflective or descriptive approach (*n* = 105). Among studies using structured methods, qualitative methods including interviews (*n* = 69, 30.1%), ethnography (*n* = 21), and focus groups (*n* = 17) were most frequently employed to understand experiences and processes. However, only one study was explicitly described as a process evaluation [17]. Fewer studies used quantitative approaches such as cross-sectional surveys (*n* = 39, 17.0%), quasi-experimental designs (*n* = 6), or randomised controlled trials (RCT) (*n* = 2). Among the RCTs, the first was a stepped-wedge cluster-randomised trial involving a multifaceted intervention to boost health policy agencies’ research engagement, including assigning knowledge brokers as project support officers for research tasks [18]. The second was a pilot RCT establishing dyads of field instructors and graduate social work students to work collaboratively on mutually beneficial, evidence-based projects [19]. However, this second example involved a ‘weaker’ form of embeddedness of a shorter duration without clear evidence of physical co-location. The methodological characteristics of the studies included in the map illustrate the challenge inherent in evaluating embedded researcher interventions and synthesising evidence in this area.

### What variations can we see in terms of how researchers were embedded and the direction of embeddedness?

Most studies confirmed an embedded researcher’s *physical co-location* (*n* = 127; 56%; Table 2). Physical co-location was reported across studies in different ways including the length of placement in a given setting (for example [20]), split of time between sites of dual-located researchers (for example [21]), or resources provided for physical co-location (for example, four studies described the importance of embedded researchers being provided with a specific desk [22–25]). Another group of studies (*n* = 23) suggested but did not confirm co-location [26]; for example, one study described a scheme where embedded researchers reported as having varying degrees of contact with their host workplaces [26].

**Table 2:** Type of embeddedness (rows) by direction of embeddedness (columns)

	Researcher to policy/professional environment	Policy/practitioner to research environment	Other
Physical co-location (confirmed)	104	29	13
Culturally/Institutionally	54	30	8
Procedurally	49	14	8
Physical co-location (probable)	17	7	2
Confirmed as embedded, nature not described	21	5	4
Embeddedness sought but not achieved	6	0	1

Note-numbers represent the number of studies; studies can feature in more than one cell

Several studies (*n* = 83) were identified where researchers were embedded *culturally or institutionally*. These described researchers being integrated into host settings and teams, for example being referred to as team members or where researchers felt like 'insiders' or ‘alongsiders’, and/or where institutional barriers were addressed (e.g. through joint contracts or oversight arrangements) so that embedded researchers were fully integrated into the host culture. We also identified instances where embedded researchers were described as being embedded in *procedural* terms (*n* = 62) such as through attending meetings, undertaking specific functions or roles within an organisation (e.g. a tutor, mentor, or support role), or where there were references to embeddedness being sought through regular communications (calls, emails, etc.). Some of the studies that *only* reported forms of procedural embeddedness may be considered those where the embeddedness was weakest. For instance, Hackett and Rhoten [27, p824] describe how “for the past 2 years we both have worked on temporary assignment at NSF [National Science Foundation], with responsibility to manage a research programme, direct a division, develop new research solicitations, serve on NSF policy committees, and conduct our research. No longer merely engaged with our research material and subjects, we had become transient government officials embedded within it”. While embeddedness was confirmed in this example, and the embedded researchers tasked with specific responsibilities (procedural embeddedness), physical co-location was not confirmed. Additional studies were identified where embeddedness was confirmed albeit with an unspecified nature (*n* = 28) or revealed attempts at becoming embedded that were not successful (*n* = 7) (see Supplementary Materials, Appendix 9 for further explanation of different types of embeddedness).

Most of the map was comprised of studies on academic researchers becoming embedded in policy or practice settings (*n* = 143, 62%). However, it also included studies reporting on policymakers and practitioners being embedded into academic settings (*n* = 35), as well as studies reporting bidirectional embeddedness (*n* = 24). A smaller group included instances where the direction was not immediately clear (described as ‘other’ forms in Table 1), sometimes due to the blurring of ‘researcher’ and ‘practitioner’ identities in an effort to break down traditional barriers (for example [28]).

### What can the map tell us about the activities that embedded researchers undertake?

Most studies (*n* = 168, 73%) described embedded researchers conducting research in situ, although only a minority (*n* = 36) stated that this was their only function. Embedded researchers were also described as seeking to create more research active cultures through knowledge brokering activities (*n* = 123) and research facilitation activities (*n* = 85). Research facilitation was defined as setting up mechanisms for skills transfer and knowledge of opportunities and linkages to enable others to conduct the research. There is substantial overlap between these pursuits and embedded researchers often conducted a blend of research generation, research facilitation, and knowledge mobilisation. For example, in a study on embedding healthcare managers as fellows into research teams, fellows undertook a mixture of duties including knowledge transfer and exchange (knowledge brokering), co-production of knowledge (research generation), and becoming a link point between academic and practice communities (an example of research facilitation) [29] (see Table 3). Studies varied in scale including evaluating a single scheme in one setting (*n* = 115) or across multiple settings (*n* = 74) through to different embedded researcher schemes across multiple settings (*n* = 40).

**Table 3:** Activities undertaken by embedded researchers (rows) by scale of intervention (columns)

	Single scheme/setting	Multi scheme/setting	Single scheme in different settings
Knowledge Brokering Activities	58	25	40
Research production activities	85	33	50
Research facilitation activities	37	13	35

Note-numbers represent the number of studies; studies can feature in more than one cell

The term ‘embedded researcher’ was not commonly used, appearing in only 7% of studies (*n* = 17). The studies used 108 different allied terms for similar activities and roles (and multiple terms were sometimes used within a single paper to describe the same activity), indicating the diverse terminology in this area. The non-generic terms that occurred most often in our map are included in Appendix 10 of the supplementary materials.

### What typologies of embedded researcher activity can we observe?

Although embedded researchers aimed to stimulate research cultures, they adopted various strategies and interventions designed differently to meet these aims. We employed Latent Class Analysis (LCA) to identify typologies of embedded researcher intervention based on the activities undertaken, nature of embeddedness, and direction of embeddedness. These factors could create 121 hypothetical intervention permutations. Using LCA, we reduced these permutations to a smaller number of latent (theorised) classes. We tested different models and found a four-class model to be satisfactory (see Appendix 11). Our manual assignment of studies to classes based on highest predicted probability overlapped entirely with the latent probabilities in the data.

The four classes we identified through Latent Class Analysis represent different typologies for embedded researcher activity (Table 4). The largest class, referred to as a ‘classic’ embedded researcher model (class 4), accounts for 45.4% of studies in the map. This type of embedded researcher intervention mainly involves researchers from research (primarily academic) institutions embedded into policy/practice settings, with researchers physically based in new settings and conducting in situ research. However, unlike other definitions for embedded researchers examined earlier, which focus on research production as the exclusive activity, this model also includes knowledge brokering activities (60.6% of studies (*n* = 104)) and/or research facilitation (31.7% of studies (*n* = 104)). Among a small minority of studies assigned to the ‘classic’ embedded researcher model, activities consisted solely of knowledge mobilisation (15.4% (*n* = 16)) or research facilitation (4.8% (*n* = 5)). Compared to classes 2 and 3, studies in this class were less likely to have been conducted within clinical settings, with greater representation of other sectors including public health and commercial and manufacturing settings (Table 5). A representative study from this class is by Bussu, Lalani and colleagues [30–33], evaluating a researcher-in-residence intervention at the intersection of health and social care. Here, embedded researchers were physically embedded within teams and focused on research co-production and knowledge mobilisation. However, this study also exemplifies the difficulties in identifying embedded researcher interventions and operationalising our criteria. For example, while we assume that embedded researchers adopt a dual affiliation as part of the ‘intervention’, this is inferred for the most part. Similarly, although we expect embedded researcher interventions to be time-bound and of an intensity sufficient to support ‘embeddedness’, the parameters around this are not described in granularity.

**Table 4:** Latent class analysis — typologies of embedded researcher in the data

	***Class 1: remote embedded researcher model***		***Class 2: low level embeddedness model***		***Class 3: reverse embedded researcher model***		***Class 4: classic embedded researcher model***	
	**Marginal mean**	**Standard error**	**Marginal mean**	**Standard error**	**Marginal mean**	**Standard error**	**Marginal mean**	**Standard error**
***Type of embeddedness***								
* Physically embedded—definite*	0.0%	0.000	0.0%	0.000	45.1%	0.070	100.0%	0.000
* Physically embedded—probable*	37.0%	0.071	0.0%	0.000	11.8%	0.045	0.0%	0.000
* Cultural or institutionally embedded*	36.9%	0.071	3.6%	0.035	56.9%	0.069	34.6%	0.047
* Procedurally embedded*	52.2%	0.074	0.0%	0.000	25.5%	0.061	24.0%	0.042
* Nature of embeddedness unclear*	0.0%	0.000	100.0%	0.001	0.0%	0.000	0.0%	0.000
***Direction of embeddedness***								
* Research to policy*	100.0%	0.000	75.0%	0.082	0.0%	0.000	100.0%	0.000
* Policy to research*	13.0%	0.050	17.9%	0.072	64.7%	0.067	15.4%	0.035
* Other direction*	4.3%	0.030	14.3%	0.066	37.2%	0.068	1.9%	0.013
***Nature of activities***								
* Research production*	71.7%	0.066	64.3%	0.091	78.4%	0.058	74.0%	0.043
* Research facilitation*	47.8%	0.074	42.8%	0.094	35.3%	0.067	31.7%	0.046
* Knowledge brokering*	50.0%	0.074	46.4%	0.094	47.1%	0.070	60.6%	0.048
* Probability in the data*	20.0%	0.026	12.2%	0.022	22.3%	0.028	45.4%	0.033
* Assigned studies*	20.1%	46	12.2%	28	22.3%	51	45.4%	104

**Table 5:** Latent class analysis — further characteristics of typologies of embedded researcher in the data

	***Class 1: remote embedded researcher model***		***Class 2: low level embeddedness model***		***Class 3: reverse embedded researcher model***		***Class 4: classic embedded researcher model***	
	**Term**	**Number and percentage**	**Term**	**Number and percentage**	**Term**	**Number and percentage**	**Term**	**Number and percentage**
**Top 3 most common terms used to describe activity**	By original profession (no new term)	5 (10.8%)	Researcher	5 (17.9%)	By original profession (no new term)	9 (17.6%)	Knowledge transfer partnership associate	14 (13.5%)
	Knowledge broker	4 (8.7%)	By original profession (no new term)	5 (17.9%)	Fellow	4 (7.8%)	Embedded researcher	10 (9.6%)
	Embedded researcher/mentor/researcher	All 2 (4.4%)			Secondment	3 (5.9%)	Researcher	10 (9.6%)
**Top 3 most common countries activity took place within**	USA	10 (21.7%)	USA	6 (21.4%)	England	14 (27.5%)	England	32 (30.7%)
	Australia	8 (17.4%)	Australia	5 (17.9%)	USA	12 (23.5%)	USA	21 (20.2%)
	England	5 (10.9%)	England	3 (10.7%)	Canada	4 (7.8%)	Canada	10 (9.6%)
**Top 3 most common sectors activity took place within**	Clinical health	11 (23.9%)	Clinical Health	9 (32.1%)	Clinical Health	30 (58.8%)	Clinical Health	28 (26.9%)
	Industry	9 (19.6%)	Social care	4 (14.3%)	Education	8 (15.7%)	Public health	19 (18.3%)
	Public health	6 (13.0%)	Public health	3 (10.7%)	Industry	4 (7.8%)	Industry	17 (16.4%)
**Total number**	46	20.10%	28	12.20%	51	22.30%	104	45.40%

The second largest class (class 3: Reverse Embedded Researcher Model) which accounted for a fifth of studies (22%; *n* = 51) does not correspond with traditional definitions of embedded researchers given that it commonly involves practitioners or policymakers embedding in academic settings or, alternatively, a blurring of identities between practitioners or policymakers and researchers. Research production is common in this class, almost half of the studies (45.1%) reported physical co-location, and over half (56.9%) exhibited cultural or institutional embeddedness. Most studies allocated to this class evaluated embedded research in clinical health (58.8%) or education (15.7%) (Table 5). An example study allocated to this class conducted by Osborne and colleagues [34] reported on ‘practitioner researchers’ who described their experiences as having ‘[been] “entangled” within the spaces between research, policy and practice in various ways’ (p207). This class signifies the importance of ‘reverse direction’ in our conception of 'embedded researchers' and highlights how research skills and ‘'fluency’ exist both within and outside academia.

The third class (*n* = 46) consists of studies with low levels of physical co-location, aligning with the shift towards online or hybrid work in recent years. This ‘physically remote’ model (class 2) had high levels of ‘procedural embeddedness’ and cultural or institutional embeddedness across sectors. All the studies allocated to this class involved researchers working in academic institutions becoming embedded ‘remotely’ in policy or practice institutions (with some bi-directionality). Despite this remote embeddedness, researchers performed a variety of duties involving research production, facilitation, and knowledge exchange. In some studies, it was noted that the absence of physical co-location was not perceived as a hindrance to team collaboration. For example, in a study conducted by Buckley and colleagues [35], a participant in a host organisation reflected: ‘there was that fear that the distance would make it difficult for us to be able to do it. And I never felt the distance, we never—it was like [Embedded Researcher] was there with us. You know?’ (p57).

The smallest class identified was a group of 28 studies (12.2%) where there were low levels of embeddedness. While embeddedness was confirmed in these studies, its nature was not fully described. There were lower levels of research production than in other classes. This group also had higher levels of variation in job titles, with many simply referred to as ‘researcher’ or by their original profession (Table 5). A study belonging to this class by Tran and colleagues [28] shows a blurring of roles between implementers and investigators in a programme of embedded implementation research, so that embedded researchers developed an ‘insider’ perspective. However, granular details on the processes of becoming embedded are left unspecified. This class underscores the challenges of identifying embedded researcher activities due to intervention heterogeneity and the complexity of reporting embeddedness in detail.

## Conclusions

### Discussion

Embedded researchers aim to bridge the gap between the production and consumption of academic research. They are aligned with broader movements to reshape the evidence ecosystem to one based on deeper collaboration between all stakeholders [36]. Embedded researcher interventions are thought to be particularly helpful in stimulating research activity in settings where the demand for research is low. Closer collaboration between generators and consumers of research is expected to create efficiencies in the flow of evidence to decision-making. Given the growing interest in bridging the research production and usage gap, it is unsurprising that over half of the evidence within this map (*n* = 118; 51.5%) was published after 2016. Our results also highlight the heterogeneity in embedded researcher ‘interventions’, and instead of providing a bridge between two distinct spheres (academia and policy/practice), the evidence generated here suggests that embedded researcher activities often blur these boundaries altogether.

However, despite the map illustrating heterogeneity in embedded researcher interventions, a limitation of our work is that we have only captured part of this variation. Our decision to limit the scope of the map to researchers who are embedded into organisations means that we have not captured instances where researchers are embedded into community settings where there is no defined organisation, but where they may be working with the purpose of creating research active communities (e.g. through co-production of research). Inclusion of these studies could reveal new models of embedded research and highlight different processes through which a more research active culture is achieved.

#### Comparison with previous literature

This map builds upon previous reviews of embedded researcher interventions (for example [1, 8]). Rather than attempting to impose a crisp definition, we have understood ‘embedded researchers’ as encompassing a set of principles. This approach allowed us to identify 108 different descriptors/allied terms for the role of an embedded researcher and resulted in a broad map of embedded researcher activities that covers a wide spectrum of approaches.

Our typologies help to illustrate a continuum of embeddedness, which can range from weak to intense and vary across physical, cultural, institutional, and procedural forms of embeddedness. These typologies also show that some models may be more emblematic of forms of co-production, for example through disrupting traditional hierarchies of power in research production and forming close and lasting relationships between research generators and consumers [37]. Many examples of embedded researcher interventions contained within the map appear to distort the boundaries between academia and policy or practice, reflecting a shift from a linear approach to a more co-productive one where research and policy or practice are intertwined.

#### Gaps in the literature

This map highlights that embedded research interventions occupy a broader suite of models than previously acknowledged. Much of the evidence base, however, is drawn from the reflections of embedded researchers, with over half of the studies in the map coded as including the reflections of researchers (as well as other stakeholders in some cases). While data based on reflections are traditionally regarded as inherently biased in terms of what is reported, the value of reflective data is increasingly being recognised [38]. Such data offer rich insights into the embeddedness process, barriers, and facilitators, and can be synthesised using techniques such as Intervention Component Analysis (ICA) [39]. The large number of studies that draw on reflections also emphasise that an ‘embedded researcher’ is a form of intervention that is both relational and subjective in nature, involving changing identities and relationships, and notions of insider/outsider status that may differ among those involved and at different points of the intervention. The map also demonstrates that few have attempted to explore the influence of embedded researchers using counterfactual evaluation approaches; for example, just two of the 229 studies utilised an RCT design.

Across the body of literature, there is a need to:

(i) Synthesise reflective evidence to understand successful strategies for becoming embedded.
(ii) Develop a more comprehensive understanding of programme theory, including better conceptualisation of outcome measurements, for different forms of embedded researcher activity.
(iii) Use the learning from (i) and (ii) to better measure the influence of embedded researcher interventions in catalysing organisations to become more research active.

The map did not reveal a clear sector- or discipline-specific embedded researcher model. Nevertheless, examining various sectors reveals different intervention designs and management approaches. An example is the UK's Knowledge Transfer Partnership scheme that involves the placement of a jointly managed embedded researcher into commercial and managerial settings (for example [40]). These partnerships reflect a long-standing structured scheme with a clear funding and management model and a clear set of objectives. This is in contrast with the more organic set-up that other embedded researchers must negotiate, which can mean that in addition to generating research activity and increasing organisational research capacity, they also need to spend time understanding and defining their own role and obtaining organisational buy-in.

## Conclusions

In producing the report of this map, we have referred to embedded researchers as a form of ‘intervention’, although have done so hesitantly. Rychentnik [41] defines an ‘intervention as comprising an action or a programme that aims to bring about identifiable outcomes’ (p540). While it is expected that the action of a researcher, policymaker, or practitioner spending time in a different environment may lead to some identifiable changes in research activity, the nature and sustainability of these expected outcomes is unclear, particularly given the heterogeneity in the model. This map highlights a clear potential for further synthesis to understand how embeddedness is achieved and to identify the possible benefits of embedding researchers. Recent contributions to the literature, including primary studies published since the main searches were carried out, also support this; they have suggested a need for further understanding of the diversity of embedded researcher schemes [4] and that embedded researchers may be in a unique position to shape and generate contextually sensitive research [42]. Given the increasing interest in this area, a better understanding of what identifiable changes could lead from embedding researchers will help to strengthen the model as well as to remove the hesitancy about regarding embedded researchers as a form of organisational level intervention.

### Supplementary Information

**Additional file 1:Appendix 1.** Steps taken in designing the search strategy and additional (non-database) searches and example database search. **Appendix 2.** Detailed inclusion/exclusion criteria. **Appendix 3. **Further details on identification of evidence. **Appendix 4.** Quality Assurance. **Appendix 5.** List of included studies and additional papers. **Appendix 6.** Distribution of studies by country. **Appendix 7.** Full list of sectors represented. **Appendix 8.** Evaluation designs implemented. **Appendix 9.** Codes and definitions for nature of embeddedness. **Appendix 10.** Terms used to describe embedded researcher interventions. **Appendix 11.**

## Data Availability

Data sharing is not applicable to this article as no datasets were generated or analysed during the current study; all data are based on articles already within the public domain. Further details of the map are available here: https://eppi.ioe.ac.uk/cms/Default.aspx?tabid=3834.
