# Supplementary material for: The implementation of embedded researchers in policy, public services, and commercial settings: a systematic evidence and gap map
Source: Implement Sci Commun. 2024 Apr 16;5:41. doi: 10.1186/s43058-024-00570-3 (PMC11020794; doi:10.1186/s43058-024-00570-3)
Supplement: Supplementary file 1 — Additional file 1:Appendix 1. Steps taken in designing the search strategy and additional (non-database) searches and example database search. Appendix 2. Detailed inclusion/exclusion criteria. Appendix 3. Further details on identification of evidence. Appendix 4. Quality Assurance. Appendix 5. List of included studies and additional papers. Appendix 6. Distribution of studies by country. Appendix 7. Full list of sectors represented. Appendix 8. Evaluation designs implemented. Appendix 9. Codes and definitions for nature of embeddedness. Appendix 10. Terms used to describe embedded researcher interventions. Appendix 11. [file 43058_2024_570_MOESM1_ESM.docx]

# Supplementary File

## Appendix 1 – Steps taken in designing the search strategy and additional (non-database) searches and example database search

In designing the search strategy, iterative and targeted searches were undertaken on Google Scholar, PubMed, and the Social Sciences Citation Index (SSCI) and browsing the journals Evidence and Policy and Research for All. SSCI searches focused on specific years (2019 and 1990) and included browsing results from key journals: (Evidence and Policy, Health Research Policy and Systems, Implementation Science). The results were scanned by title and the most relevant 154 were screened on title and abstract by the review team (DK, SL and CS). Out of these 60 looked potentially relevant and were used to develop and test the search strategy and inform additional search terms, particularly within SSCI. We drew on the work of previous reviews and search methods in knowledge translation, including: [13-16]. We also asked the advisory group if they had any useful insights on terminology and sources of research.

### Website searches

The following websites were checked during March 2021: Alliance for Useful Evidence, CanChild, CAPE, CLARHC/ ARC, UK PRP, Fuse, Transforming Evidence, Evaluation Support Scotland, Michael Smith Foundation for Health Research, KT Pathways, Universities Policy Engagement Network (UPEN), WT Grant Foundation, KTDRR, Rethinking Research Collaborative

### Other searching methods

The titles of articles published in the journals Evidence and Policy and Research for All were scanned up until May 2021. Related relevant references were also identified ad-hoc as a result of undertaking full-text screening, and 154 references from the scoping exercise were re-screened for relevance.

Search History used in Web of Science Databases

Indexes=SCI-EXPANDED, SSCI, BKCI-S, BKCI-SSH, ESCI Timespan=1991-2021

19/5/2021, 4,599 records

# 34 (#33)  AND LANGUAGE: (English)

# 33 #32 AND #31

**Study design terms** # 32 TS=("impact" OR effective* OR evaluat* OR "lesson learned" OR "case study" OR "case studies" OR "feasibility study" OR "pilot study" OR "feasibility studies" OR "pilot studies" OR "experiences" OR "qualitative*" OR "perspective*" OR "interviews" OR "findings" OR "focus group" OR "focus groups" OR "outcome*" OR "performance measure*" OR "performance assessment" OR "mixed methods" OR "proof of concept" OR (critical NEAR/2 (examination* OR apprais* OR reflect*)  ) OR "programme effect*" OR "program effect*" OR "observed effects" OR "observational study" OR "programme impact*" OR "program impact*" OR "observed effect*" OR "observations" OR "survey" OR "surveys" OR "questionnaire*" OR "feedback" OR "model" OR "models" OR framework OR frameworks)  OR TI=("analysis" OR study*)

# 31 #30 OR #29

# 30 #27 AND (#26 OR #25) AND ( #1 OR #2)

# 29 #28 AND #24

# 28 (#27 AND #26) OR #25

**Context terms** # 27 (TS=(( ((policy OR practice OR evidence) NEAR/2 interface*) OR ((evidence OR policy) NEAR/1 practice) OR ("evidence informed decision*" OR EIDM OR "evidence-based decision*" OR EBM OR "evidence-based medicine" OR "Evidence-Based Practice*" OR "Evidence-informed Practice" OR "Evidence based policy*" OR "Evidence informed policy*" OR "policy and practice" OR "Policy Making" OR "Policymaking") OR (("evidence" OR "knowledge") NEAR/3 (utilis* OR utiliz* OR uptake) AND (decision* OR policy*) )) OR ("knowledge to action" OR "research use" OR "evidence use" OR "use research" OR "use evidence" OR (diffusion NEAR/1 innovation) OR (evidence NEAR/2 (diffusing or diffusion or exchange or implement* OR transfer* OR translat* OR uptak* OR utiliz* OR mobil* OR broker*) ) OR (implementation NEAR/3 research) OR "implementation science" OR (innovation* NEAR/2 (adopt* OR diffusion OR implement*) ) OR "integrated knowledge transfer" OR "integrated knowledge transformation" OR "integrated knowledge translation" OR (Knowledge NEAR/2 (adopt* OR broker* OR diffusion OR integration OR mobil* OR exchang* OR transform* OR transfer* OR translat* OR uptak* OR utiliz* OR engag*) ) OR (("Mode two knowledge" or "Mode II knowledge" or "Mode 2 knowledge") NEAR/2 (produc* OR generat* OR creat* OR develop*) ) OR (("Mode two research" or "Mode II research" or "Mode 2 research") NEAR/2 (produc* OR generat* OR creat* OR develop*) ) OR (research NEAR/2 (broker* OR diffusion OR integration OR mobil* OR exchang* OR transfer* OR translat* OR uptak* OR utiliz* OR transformation OR implement*) ) OR (science NEAR/2 (implement* OR utilizat*) ) OR (technology NEAR/2 transfer) )))

**Context terms** # 26 (TS=("regional authority" OR "regional government" OR "local authority" OR "local authorities" OR "service organisation*" OR "service organization*" OR "local government*" OR ((government* OR community OR policy OR "non academic") NEAR/2 organisation) OR ((government* OR community OR policy OR "non academic") NEAR/2 organisations) OR ((government* OR community OR policy OR "non academic") NEAR/2 organization) OR ((government* OR community OR policy OR "non academic") NEAR/2 organizations) OR ((government* OR community OR policy OR "non academic") NEAR/2 service) OR ((government* OR community OR policy OR "non academic") NEAR/2 setting) OR ((government* OR community OR policy OR "non academic") NEAR/2 agency) OR "non governmental" OR "third sector" OR "non academic setting" OR "government department" OR "government unit" OR (professional NEAR/2 association) OR (professional NEAR/2 body) OR (professional NEAR/2 institut*) OR "commercial partner*" OR "commercial organi*" OR "private partner*" OR "private organi*" OR "trade association*" OR "research association" OR "research associations" OR "technical information service*" OR (technical NEAR/2 (centre OR centres OR center OR centers) ) OR industry OR (service* NEAR/2 provider*) ))

**Context terms** # 25 (TS=((health* NEAR/2 service*) OR (health* NEAR/2 system) OR (health* NEAR/2 delivery) OR (practitioner* OR commissioner*) OR (health* NEAR/2 worker*) OR (health* NEAR/2 workforce) OR (health* NEAR/2 "work force") OR (health* NEAR/2 systems) OR (public NEAR/2 health*) OR (health NEAR/2 promot*) OR (health NEAR/2 educat*) OR (health* NEAR/2 intervention*) OR "primary prevention" OR " preventive care" OR "preventive health*" OR "primary health*" OR "primary care" OR "preventive medicine" OR "community health*" OR "healthcare" OR "health care" OR "social care" OR "social service*" OR "social work*" OR (care NEAR/2 (system OR systems OR service* OR community OR integrated OR setting* OR centre OR center OR centres OR centers) ) OR "healthcare trust" OR "health care trust" OR NHS OR "secondary care trust" OR CLAHRC OR (health* NEAR/2 department) OR (health* NEAR/2 government) ))

# 24 #23 OR #22 OR #21 OR #20 OR #19 OR #18 OR #17 OR #16 OR #15 OR #14 OR #13 OR #12 OR #11 OR #10 OR #9 OR #8 OR #7 OR #6 OR #5 OR #4 OR #3

**Intervention terms:**

# 23 TS=(("co-creat*" OR cocreat* OR "co-produc*" OR coproduc*) NEAR/2 (evidence OR knowledge OR research) ) OR TS=((cocreat* OR coproduc* OR "co-creat*" OR "co-produc*" OR "roles") NEAR/10 ("technical assistance" OR "technical support" OR (change NEAR/1 facilita*) OR (change NEAR/1 agent*) OR (capacity NEAR/2 (build* OR strength* OR develop*) )))

# 22 TS=(("intermediary organisation*" OR "intermediary organization*") NEAR/3 (practice OR policy OR practice OR evidence OR decision* OR EIDM OR EBM OR "Policymaking" OR innovation OR (technology NEAR/2 transfer) ))

# 21 TS=("co-locate" OR "co-location" OR "co-locating" OR secondment* OR ("boundary" NEAR/2 ("span" OR "spanning" OR "spanner" OR "spanners" OR "bridging" OR "bridger*") ) OR "Liaison research*" )

# 20 TS=((broker* OR exchange* OR translation OR mobilis* OR mobiliz*) NEAR/2 (research* OR academic*)   NEAR/3 (practice OR policy OR practice OR evidence OR decision* OR EIDM OR EBM OR "Policymaking" OR innovation Or (technology NEAR/2 transfer) ))

# 19 TS=(((Partner* OR broker* OR intermediaries OR "technical support" OR "technical assistance" OR collaborator* OR "collaborative working" OR "roles") NEAR/10 ((change NEAR/1 facilita*) OR (change NEAR/1 agent*) OR (capacity NEAR/2 (build* OR strength* OR develop*) ) OR "knowledge exchange*" OR "knowledge broker*" OR "knowledge transfer*" OR "knowledge mobilis*" OR "knowledge mobiliz*" OR "implementation scien*" OR "implementation research" OR mentor* OR coach*)) NEAR/10 (practice OR policy OR practice OR evidence OR decision* OR EIDM OR EBM OR "Policymaking" OR innovation OR (technology NEAR/2 transfer) ))

# 18 TS=((program* OR initiativ* OR strategy OR strategies* OR infrastruct*) NEAR/10 ((change NEAR/1 facilita*) OR (change NEAR/1 agent*) OR (capacity NEAR/2 (build* OR strength* OR develop*) ) OR "knowledge exchange*" OR "knowledge broker*" OR "knowledge transfer*" OR "knowledge mobilis*" OR "knowledge mobiliz*" OR "implementation scien*" OR "implementation research" OR mentor OR coach*) NEAR/10 (practice OR policy OR practice OR evidence OR decision* OR EIDM OR EBM OR "Policymaking" OR innovation OR (technology NEAR/2 transfer) ))

# 17 TS=((research* OR university* OR academic* OR academia*) NEAR/5 (partner*) NEAR/5 (broker*) )

# 16 TS=(("co-locate" OR "co-location" OR "co-locating" OR secondment*) NEAR/3 research*)

# 15 TS=((policy* NEAR/1 fellow*) OR (policy* NEAR/2 research* NEAR/2 fellow*) )

# 14 TS=((embedded* OR collaborative* OR intermediaries* OR roles)  NEAR/5 (evidence OR knowledge ) NEAR/3 (broker* OR exchange* OR transfer* OR translation OR mobilis* OR mobiliz* OR transform*) ) OR TS=((embedded* OR collaborativ* OR intermediar*) NEAR/5 ("implementation research*" OR "implementation scien*") )

# 13 TS=("integrated knowledge exchange" OR "integrated knowledge mobili*" OR "integrated knowledge translation" OR "integrated knowledge transfer*") OR

TS=("knowledge intermediaries")

# 12 TS=(("researcher*" OR "research team" OR "research group" OR "research groups" OR professor* OR "doctoral student" OR "research fellow" OR "research fellows" OR "postdoctoral fellow" OR "postdoctoral fellows" OR "research associate" OR "research associates" OR "research staff" OR "scholar" OR "scholars" OR "academics" OR "academic fellow" OR "academic staff")  NEAR/3 (embedded* OR secondment* OR "seconded" OR "liaison" OR "adjunct" OR intermediar* OR "brokers" OR "broker" OR mediator* OR "co-locate" OR "co-location" OR "co-locating") )

# 11 TS=((academic* OR "researcher*" OR "research team" OR "research group" OR "research groups" OR professor* OR "doctoral student" OR "research fellow" OR "research fellows"

OR "postdoctoral fellow" OR "postdoctoral fellows" OR "research associate" OR "research associates" OR "research staff" OR "scholar" OR "scholars" OR "academics" OR "academic fellow" OR "academic staff")) AND TS=(("policy placement*")OR("project placement*"))

# 10 TS=(("researcher*" OR "research team" OR "research group" OR "research groups" OR professor* OR "doctoral student" OR "research fellow" OR "research fellows" OR "doctoral fellow" OR "doctoral fellows" OR "postdoctoral fellow" OR "postdoctoral fellows" OR "post doctoral fellow" OR "post doctoral fellows" OR "research associate" OR "research associates" OR "research staff" OR "scholar" OR "scholars" OR "academics" OR "academic fellow" OR "academic staff") NEAR/5 (role OR roles) ) AND TS=((change NEAR/1 facilita*) OR (change NEAR/1 agent*) OR (capacity NEAR/2 (build* OR strength* OR develop*) ) OR "knowledge exchange*" OR "knowledge broker*" OR "knowledge transfer*" OR "knowledge mobilis*" OR "knowledge mobiliz*" OR "implementation scien*" OR "implementation research" OR mentor* OR coach*)

# 9 TS=(("researcher" OR "scholar" OR "scholars" OR "academic" OR "academics" or "professor*") NEAR/2 ("residence" OR "resident" OR "broker" OR "brokers") )

# 8 TS=(("champion" OR "champions" or "broker" OR "brokers") NEAR/1 (research or academic) )

# 7 TS=(("co-locate" OR "co-location" OR "co-locating" OR encultur* OR "credible insider" OR "credible insiders" OR secondment*) NEAR/10 ((change NEAR/1 facilita*) OR (change NEAR/1 agent*) OR (capacity NEAR/2 (build* OR strength* OR develop*) ) OR "knowledge exchange*" OR "knowledge broker*" OR "knowledge transfer*" OR "knowledge mobilis*" OR "knowledge mobiliz*" OR "implementation scien*" OR "implementation research" OR mentor* OR coach*) )

# 6 TS=(("boundary" NEAR/2 ("span" OR "spanning" OR "spanner" OR "spanners" OR "bridging" OR "bridger*") ) OR "Liaison research*")

# 5 TS=((organisation* OR organization* OR institution*) NEAR/2 (program* OR initiativ* OR strategy OR strategies* OR infrastruct* OR mentor* OR coach*) ) AND TS=(((change NEAR/1 facilita*) OR (change NEAR/1 agent*) OR (capacity NEAR/2 (build* OR strength* OR develop*) ) OR "knowledge exchange*" OR "knowledge broker*" OR "knowledge transfer*" OR "knowledge mobilis*" OR "knowledge mobiliz*" OR "implementation scien*" OR "implementation research"))

# 4 TS=(("researcher*" OR "research team" OR "research group" OR "research groups" OR professor* OR "doctoral student" OR "research fellow" OR "research fellows" OR "doctoral fellow" OR "doctoral fellows" OR "postdoctoral fellow" OR "postdoctoral fellows" OR "post doctoral fellow" OR "post doctoral fellows" OR "research associate" OR "research associates" OR "research staff" OR "scholar" OR "scholars" OR "academics" OR "academic fellow" OR "academic staff") NEAR/3 ((change NEAR/1 facilita*) OR (change NEAR/1 agent*) OR (capacity NEAR/2 (build* OR strength* OR develop*) ) OR "knowledge exchange*" OR "knowledge broker*" OR "knowledge transfer*" OR "knowledge mobilis*" OR "knowledge mobiliz*" OR "implementation scien*" OR "implementation research" OR mentor* OR coach*) )

# 3 TS=(("researcher*" OR "research team" OR "research group" OR "research groups" OR professor* OR "doctoral student" OR "research fellow" OR "research fellows" OR "doctoral fellow" OR "doctoral fellows" OR "postdoctoral fellow" OR "postdoctoral fellows" OR "post doctoral fellow" OR "post doctoral fellows" OR "research associate" OR "research associates" OR "research staff" OR "scholar" OR "scholars" OR "academics" OR "academic fellow" OR "academic staff")  AND ("co-locate" OR "co-location" OR "co-locating" OR encultur* OR "credible insider" OR "credible insiders" OR secondment*) )

# 2 TS=((Partner* OR broker* OR "intermediaries" OR "technical support" OR" technical assistance" OR collaborator* OR "collaborative working" OR "roles") NEAR/5 (("change facilitator" OR mentor* OR (change NEAR/1 facilita*) OR (change NEAR/1 agent*) OR (capacity NEAR/2 (build* OR strength* OR develop*) ) OR coach* OR "knowledge exchange*" OR "knowledge broker*" OR "knowledge transfer*" OR "knowledge mobilis*" OR "knowledge mobiliz*" OR "implementation scien*" OR "implementation research")))

# 1 TS=(("researcher*" OR "research team" OR "research group" OR "research groups" OR professor* OR "doctoral student" OR "research fellow" OR "research fellows" OR "doctoral fellow" OR "doctoral fellows" OR "post-doctoral fellow" OR "post doctoral fellows" OR "postdoctoral fellow" OR "postdoctoral fellows" OR "research associate" OR "research associates" OR "research staff" OR "scholar" OR "scholars" OR "academics" OR "academic") NEAR/2 (Partner* OR broker* OR intermediaries OR "technical support" OR "technical assistance" OR collaborator* OR "collaborative working") )

## Appendix 2 – Detailed inclusion/exclusion criteria

- Exclude - Duplicate
- Exclude - Activity not centred around enabling organisations to utilize, commission, or undertake research
- Exclude – Study does not present any empirical data or descriptions including from case studies e.g. study is a commentary or call to action
- Exclude – The activity is not focussed on enabling research or research use within policy, practice or commercial formal organisations (for example the study evaluated measures to enhance the flow of evidence in general/abstract terms without defined target)
- Exclude - The researcher(s) is not situated within a defined organization (for example the researcher is embedded within a community coalition without a clear organisation)
- Exclude – The researcher(s) is not situated within host team (physically or institutionally or affiliation or culturally). The researcher(s) is expected to be situated within a host team (physically or institutionally or affiliation or culturally) and/or expected to work within the host team culture for a high proportion of their time
  - Cultural or institutional embeddedness included references to being described as a team member in host team; being embedded in team culture; being set up to be a staff member of host institution (e.g. through holding a joint contract or contract funded by host org – i.e. institutional barriers removed); to being made to feel welcome/integrated etc.; to being viewed as fully participating in host organisation’s daily practice; and references to transdisciplinarity
  - Procedural embeddedness included references to specific procedures/processes that may be viewed as a form of embeddedness - for example references to regularly attending staff meetings in host organization, conducting specific tasks or occupying specific roles (e.g. tutor role or support role) within the host organization; and references to embeddedness being sought through regular emails, calls etc.
- Exclude – The researcher(s) has no ongoing affiliation with or specific funding from a research organisation
- Exclude – The initiative is taking place without an explicit aim of enabling research activity or capacity
- Exclude – The researcher(s) does not have active and continued engagement with host team
- Exclude – The researcher(s) does not have longer-term affiliation/placement (exclude if the placement is shorter than a month); exclude if the embedded researcher(s) is a permanent member of staff; or the embedded researcher activity is not time-limited
- Exclude – The host organisation is not able to direct or influence the research/activities of the researcher i*.*e. research is conducted within an organisation without that organisation being able to influence the activity (e.g. an ethnography of the organization)
- Exclude – The researcher is undertaking placement for taught degree (not a research degree)
- Exclude - No indication of two-way relationship and two-way organisational learning
- Exclude – Exclude if the study is meta study and describes the evaluation of wide programmes of activity where embedded research may be part of remit but the study is not a focussed evaluation of embedded researcher activity (NB: for example embedded researcher schemes that were reported as part of broader Academic Collaborative Centres (see Molleman and Fransen 2012 for an overview of ACCs) and Collaborations for Leadership in Applied Health Research and Care (see Rycroft-Malone et al. 2011 for an overview of CLAHRCs) were excluded on this basis).
- Exclude – Study reports on a placement between academic/research organisations or between policy/practice organisations (e.g. policy to policy setting, or from one academic discipline to another)
- Exclude – Study reports on the research produced through an embedded researcher scheme but does not evaluate the embedded researcher scheme itself
- Exclude – Researcher(s) embedded into diffuse organisations
- Exclude – Abstract only available

Studies that were not excluded based on the criteria above were included in the map.

## Appendix 3 – Further details on identification of evidence

Results from literature searches were imported into to EPPI-Reviewer [10] and duplicates removed. Reviewers examined title and abstracts for relevance initially in duplicate and then independently, having first piloted the inclusion and exclusion criteria to ensure consistency of screening decisions. Piloting started through screening a small sample of 40 records initially with an agreement rate of 75% and a Kappa value of 0.65 and the team discussed disagreements; larger samples were then trialled ((n=140; agreement rate=0.95; Kappa=0.64) and (n=394; agreement rate=0.96; Kappa=0.77)) where we achieved substantial levels of agreement. Reviewers then screened the records independently, although where a decision was unclear to a reviewer, this was marked as a query for further investigation and decision by review team members in weekly meetings. The full texts of all references meeting the eligibility criteria were retrieved and checked for eligibility, and studies meeting the eligibility criteria after full-text screening were coded for the map.

 It was planned at the outset that the volume of irrelevant items manually screened against the review's inclusion criteria would be reduced by using priority screening based on text-mining to prioritise the most likely relevant studies, and that manual assessment of studies would cease once an appropriate cut-off point was determined. The machine learning reviewing software in EPPI-Reviewer ‘learns’ to recognise citations that are likely to be included and excluded based on how researchers apply screening criteria [17, 18]. Citations are sorted iteratively throughout the screening process so that those most likely to be included are screened first, prioritising these to significantly speed up the screening process [19, 20].  However, all references without abstracts, references identified from website searches and new references identified from full-text screening, were screened manually.

To implement priority screening, several steps were taken. First, a random sample of 423 citations was screened to predict an initial inclusion rate of 8.7%. Second, a power calculation (using Lenth’s [21] tool) was performed to ensure the sample size was sufficient, which indicated that the sample and predicted inclusion rate of 8.7% had a margin of error of 2.65% at a 95% conference interval. To determine when to stop screening, three pieces of information were considered. Firstly, the predicted number of eligible studies was reached based on the baseline inclusion rate. Secondly, a graph was observed to monitor the inclusion rate during the priority screening process. Thirdly, unscreened citations were ranked for relevance by a machine classifier and the highest-ranking records (with a relevance score of 20-40%) were manually screened; from this 146 title/abstract records were screened and the full-texts of eight were screened, identifying one eligible study.

As described above, title and abstract screening was first conducted in duplicate and then independently once a sufficient level of agreement had been reached. We had intended for all full text screening to also be conducted in duplicate, although the high number of records put forward for full text screening (over 1000) meant that we again undertook duplicate screening for a small batch initially ((n=40; agreement rate=0.97; Kappa=0.92)) and achieved a high level of consistency before independent screening took place. A query code was used frequently by reviewers to flag records for discussion and a second opinion before an inclusion/exclusion code was selected.

## Appendix 4 – Quality Assurance

In addition to the steps outlined above, the following measures were employed for quality assurance purposes:

1. **Exploring existing reviews** – We explored existing reviews directly on the topic of interest (i.e. specifically embedded researchers however termed by the authors), and used these to further assess the success of the search and screening processes. Any missing studies were added to the map provided they met our inclusion criteria.
2. **Use of second opinion/query codes** - Members of the coding team referred any records about which they were uncertain for a 'second opinion' – second opinion codes were used for title and abstract screening, full text screening, and for coding issues. The coding of 'second opinion' records was then resolved through the input of an additional reviewer or through wider consensus and discussion among reviewers.
3. **Checking coding reports** – All coding for studies was double checked by a second reviewer. This involved checking that the evidence highlighted by a reviewer appropriately supported the choice of code.
4. **Full text checking** – A sample of 10% of included studies was fully double coded by a second reviewer. This helped to highlight where there may be particular codes that may have been inconsistently applied.
5. **Checking problematic codes** – Based on the checks above, where a code was viewed to have been applied inconsistently across studies, further checking of that code was undertaken to verify the information associated with the code.

## Appendix 5 – List of included studies and additional papers

| **First Author and Date** | **Title** |
| --- | --- |
| Allen (2010) | A fundamental partnership: the experiences of practising teachers as lecturers in a pre-service teacher education programme |
| Ancess (2007) | Using research to inform the practice of teachers, schools, and school reform organizations |
| Artz (2017) | participation in practice: a case study of a collaborative project on sexual offences in South Africa |
| Arzensek (2014) | Slovenian young researchers' motivation for knowledge transfer |
| Asgary (2018) | A Collaborative Multidisciplinary and Without-Walls Research Curriculum in Global Health |
| Asmonga (2020) | The space between: Using inquiry teams to encourage multilevel boundary crossing between educational research and practice |
| Asthana (2020) | Capturing the Role of Context in Complex System Change: An Application of the Canadian Context and Capabilities for Integrating Care (CCIC) Framework to an Integrated Care Organisation in the UK |
| Baba (2010) | Sources of success in advanced materials innovation: the role of 'core researchers' in univerity-industry collaboration in Japan |
| Babu (2000) | Impact of IFPRI's policy research on resource allocation and food security in Bangladesh |
| Beck-Pettersson (2013) | Nurses' experiences of participation in a research and development programme |
| Bannister (2011) | Building safer communities: Knowledge mobilisation and community safety in Scotland |
| Bartelink (2019) | Facilitators and barriers to brokering between research and care by senior clinical-scientists in general practice and elderly care medicine |
| Bartram (2010) | Review of NCVER building researcher capacity initiative  **Additional papers:**  *Bowden (2015) NCVER building researcher capacity scholarship: A rural participant's perspective*  *Gutteridge (2015) Chronicle of a novice researcher: the challenges and rewards* |
| Baumbusch (2018) | Exploring the synergies between focused ethnography and integrated knowledge translation |
| Berggren (2008) | Rethinking project management education: Social twists and knowledge co-production |
| Berzin (2019) | Building Capacity for Innovation: The Case of an Academic-Nonprofit Partnership |
| Blanchette (2019) | Making Contributions and Defining Success: An eDelphi Study of the Inaugural Cohort of CIHR Health System Impact Fellows, Host Supervisors and Academic Supervisors  **Additional papers:**  *Bornstein (2019) Exploring Mentorship as a Strategy to Build Capacity and Optimize the Embedded Scientist Workforce*  *Cassidy (2019) It's All About the IKT Approach: Three Perspectives on an Embedded Research Fellowship Comment on "CIHR Health System Impact Fellows: Reflections on 'Driving Change' Within the Health System"*  *Eljiz (2020) The Embedded Health Management Academic: A Boundary Spanning Role for Enabling Knowledge Translation Comment on "CIHR Health System Impact Fellows: Reflections on 'Driving Change' Within the Health System"*  *Hunter (2019) Meeting the Challenge of the "Know-Do" Gap Comment on "CIHR Health System Impact Fellows: Reflections on 'Driving Change' Within the Health System"*  *McMahon (2019) The Health System Impact Fellowship: Perspectives From the Program Leads Comment on "CIHR Health System Impact Fellows: Reflections on 'Driving Change' Within the Health System"*  *McMahon (2019) Training for Health System Improvement: Emerging Lessons from Canadian and US Approaches to Embedded Fellowships* |
| Blannin (2020) | Teachers as embedded practitioner-researchers in innovative learning environments |
| Boccia (2019) | How to Integrate Personalized Medicine into Prevention? Recommendations from the Personalized Prevention of Chronic Diseases (PRECeDI) Consortium |
| Borrell-Damian (2010) | Collaborative Doctoral Education: University-Industry Partnerships for Enhancing Knowledge Exchange |
| Bosi (2013) | A Collaborative Project Between Industry and Academia to Enhance Engineering Education at Graduate and PhD Level in Ceramic Technology |
| Bosley (1994) | Our experience as collaborative ethnographers |
| Breu (2005) | Researcher-Practitioner Partnering in Industry-Funded Participatory Action Research |
| Brooks (2017) | Increasing Research Capacity in a Safety Net Setting Through an Academic Clinical Partnership |
| Bruce (2016) | Inside out: Knowledge brokering by short-term policy placements |
| Buckley (2021) | The relational nature of Evaluation Capacity Building: Lessons from facilitated evaluation partnerships  **Additional papers:**  *Chauveron (2021) Promoting evaluation in youth character development through enhanced Evaluation Capacity Building: Empirical findings from the PACE Project* |
| Bullock (2012) | Collaboration between health services managers and researchers: making a difference? |
| Bullock (2013) | Exchanging knowledge through healthcare manager placements in research teams |
| Bullock (2016) | Getting the most out of knowledge and innovation transfer agents in health care: a qualitative study |
| Burton (2016) | Innovative Graduate Research Education for Advancement of Implementation Science in Adolescent Behavioral Health |
| Bush (2016) | Building on a YMCA's health and physical activity promotion capacities: A case study of a researcher-organization partnership to optimize adolescent programming_ |
| Bussu (2020) | Engaging with care: ethical issues in Participatory Research  **Additional papers:**  *Bussu (2020) (Dis)Integrated Care? Lessons from East London*  *Lalani (2019) Transforming community nursing services in the UK; lessons from a participatory evaluation of the implementation of a new community nursing model in East London based on the principles of the Dutch Buurtzorg model*  *Lalani (2020) Understanding integrated care at the frontline using organisational learning theory: A participatory evaluation of multi-professional teams in East London*  *Lalani (2020b) Co-location, an enabler for service integration? Lessons from an evaluation of integrated community care teams in East London* |
| Campbell (2016) | Knowledge co-production at the researchâ€“practice interface: Embedded case studies from urban forestry |
| Carter (2020) | Evaluation of the joint nurse scientist role across academia and practice |
| Carter (2020) | Programmatic details and outcomes of an Academic-Practice Research Fellowship for clinical nurses |
| Cato (2019) | Linking to Improve Nursing Care and Knowledge: Evaluation of an Initiative to Provide Research Support to Clinical Nurses |
| Chapman (2005) | Collaboration in the Emergency Department: an innovative approach  **Additional papers:**  *Chapman (2011) Leading change and advancing health by enhancing nurses' and midwives' knowledge, ability and confidence to conduct research through a clinical scholar program in Western Australia* |
| Chappell (2008) | Mediating creativity and performativity policy tensions in dance education-based action research partnerships: Insights from a mentor's self-study |
| Cheetham (2018) | Embedded research: a promising way to create evidence-informed impact in public health?  **Additional papers:**  *Van der Graaf (2020) Mobilising knowledge in public health: Reflections on ten years of collaborative working in fuse, the centre for translational research in public health* |
| Cheetham (2019) | Local Authority Champions of Research Project: a report for the Health Foundation |
| Chew (2013) | Institutionalising knowledge brokering as a sustainable knowledge translation solution in healthcare: How can it work in practice? |
| Chiaroni (2014) | The Role of Knowledge Brokers in Open Innovation |
| Chivers (2005) | Ordinary magic: developing services for children with severe communication difficulties by engaging multiple voices |
| Christensen (2017) | The nursing professorial unit: translating acute and critical care nursing research |
| Churruca (2019) | The time has come: Embedded implementation research for health care improvement |
| Cleaver (2013) | Knowledge transfer partnerships: a case study |
| Coates (2020) | The embedded researcher model in Australian healthcare settings: comparison by degree of "embeddedness" |
| Coates (2020) | Challenges and enablers of the embedded researcher model |
| Cole (2015) | Evaluating the Development, Implementation and Dissemination of a Multisite Card Study in the WWAMI Region Practice and Research Network |
| Compton (2001) | The Collaborative Evaluation Fellows Project: background and overview of the model |
| Conrad (2013) | Participants' Perceptions of a Multidisciplinary Training Program for Graduate and Postgraduate Students in Drug Use Management and Policy |
| Cooke (2008) | An evaluation of the 'Designated Research Team' approach to building research capacity in primary care |
| Coutts (2017) | Project Oracle: The glue between researchers, commissioners and practitioners. |
| Cowdean (2019) | Entrepreneurial learning in practice: The impact of knowledge transfer |
| Crist (2005) | The practice-scholar program: an academic-practice partnership to promote the scholarship of 'best practices' |
| Crosby (2013) | The community leaders institute: an innovative program to train community leaders in health research |
| Cukurova (2019) | Creating the golden triangle of evidence-informed education technology with EDUCATE |
| Cunliffe (2017) | Embedding Impact in Engaged Research: Developing Socially Useful Knowledge through Dialogical Sensemaking |
| Cunningham (2010) | Building evaluation capacity with appreciative inquiry: An exploratory case study |
| De Ossorno (2021) | The mentoring relation as an interpersonal process in EDUCATE: A qualitative case study of mentorâ€“mentee perspectives  **Additional papers:**  *Clark-Wilson (2021) Supporting small and medium-sized enterprises in the educational technology sector to become more research-minded: Introduction to a small collection*  *Luckin (2021) What inspired my thinking to create UCL EDUCATE?* |
| Dickinson, (2017) | 2017 UK-Wide Survey of Clinical and Health Research Fellowships |
| Dobbins (2019) | A description of a tailored knowledge translation intervention delivered by knowledge brokers within public health departments in Canada |
| Donaldson (2011) | A Nurse Manager's Reflections On Adapting To A Research Role |
| Downie (2001) | A practice-research model for collaborative partnership |
| Duffy (2016) | Nurses' Research Capacity, Use of Evidence, and Research Productivity in Acute Care |
| Duggan (2014) | Critical friendship and critical orphanship: embedded research of an English local authority initiative |
| Dzotsenidze (2017) | The Contribution of the International Pain Policy Fellowship in Improving Opioid Availability in Georgia |
| Edwards (2007) | Organizational politics and the "process of knowing": Understanding crisis events during project-based innovation projects |
| Edwards (2007) | A critical account of knowledge management: agentic orientation and SME innovation |
| Edwards (2016) | The impact of leadership hubs on the uptake of evidence-informed nursing practices and workplace policies for HIV care: a quasi-experimental study in Jamaica, Kenya, Uganda and South Africa |
| Elg (2008) | Implementing statistical process control: an organizational perspective |
| Evans (2014) | Supporting knowledge translation through collaborative translational research initiatives:â€˜Bridgingâ€™versus â€˜blurringâ€™boundary-spanning approaches in the UK CLAHRC initiative |
| Eyre (2017) | What can a participatory approach to evaluation contribute to the field of integrated care?  **Additional papers:**  *Eyre (2015) Protocol for a process-oriented qualitative evaluation of the Waltham Forest and East London Collaborative (WELC) integrated care pioneer programme using the Researcher-in-Residence model* |
| Ferguson (2020) | From Snapshots of Practice to a Movie: Researching Long-Term Social Work and Child Protection by Getting as Close as Possible to Practice and Organisational Life |
| Firdaus (2020) | Community Partnership Through Knowledge Transfer Program: Assessment From the Perspectives of Academics' Experience |
| Forrester (2008) | Professor in residence program: a nursing faculty practice |
| Fry (2017) | Interventions to support and develop clinician-researcher leadership in one health district |
| Galan (2018) | "One foot in industry, the other in academia": Why professional services want adjunct professors as employees? |
| Gerrish (2014) | Capacity development for knowledge translation: evaluation of an experiential approach through secondment opportunities |
| Gertner (2011) | University-industry collaboration: a CoPs approach to KTPs |
| Goode (2018) | The McDonaldisation of police-academic partnerships: organisational and cultural barriers encountered in moving from research on police to research with police |
| Gradinger (2019) | Reflections on the Researcher-in-Residence model co-producing knowledge for action in an Integrated Care Organisation: A mixed methods case study using an impact survey and field notes  **Additional papers:**  *Gradinger (2020) Integrating the voluntary sector in personalised care: mixed methods study of the outcomes from wellbeing co-ordination for adults with complex needs* |
| Gregory (2015) | Evidence-based decision-making 5: Translational research |
| Hackett (2011) | Engaged, Embedded, Enjoined: Science and Technology Studies in the National Science Foundation |
| Hagen (2002) | Globalization, university tranformation and economic regeneration: A UK case study of public/private sector partnership |
| Hamelin (2018) | Population health intervention research training: the value of public health internships and mentorship |
| Hay (2003) | The nature and significance of the executive doctoral scholar -practitioner of organizational development and change: A morphogenetic account of theory -practice linkages for the achievement of scholarly knowledge and business results |
| Hazeldine (2021) | Link worker perspectives of early implementation of social prescribing: A 'Researcher-in-Residence' study |
| Hendrix (2011) | The Duke-NICHE program: an academic-practice collaboration to enhance geriatric nursing care |
| Henningsson (2019) | Senior Industry Practitioners as Part-Time Visiting Professors: The Various Benefits of Collaboration |
| Henry (2012) | Brokering communities of practice: A model of knowledge exchange and academic-practitioner collaboration developed in the context of community policing |
| Henshall (2021) | Interventions and strategies aimed at clinical academic pathway development for nurses in the United Kingdom: A systematised review of the literature |
| Higuchi (2017) | Knowledge sharing between academic researchers and tourism practitioners: a Japanese study of the practical value of embeddedness, trust and co-creation |
| Ho (2016) | Technology upgrading of Small-and-Medium-sized Enterprises (SMEs) through a manpower secondment strategy - A mixed-methods study of Singapore's T-Up program |
| Hoens (2013) | Knowledge brokering: an innovative model for supporting evidence-informed practice in respiratory care  **Additional papers:**  *Hoens (2013) Physical Therapy Knowledge Broker Report Year 3 September 2011 to October 2012*  *Hoens (2014) The Knowledge Broker's "Fit" in the World of Knowledge Translation* |
| Hofmeyer (2012) | Researcher-decision-maker partnerships in health services research: practical challenges, guiding principles |
| Holbrook (2017) | Building Capacity for Evidence-Based Practice Together |
| Holly (2014) | Moving evidence to practice: reflections on a multisite academic-practice partnership |
| Hope (2016) | Creating sustainable cities through knowledge exchange A case study of knowledge transfer partnerships |
| Hulcombe (2014) | An approach to building research capacity for health practitioners in a public health environment: an organisational perspective |
| Humphries (2005) | Fight past the jargon and find the benefits |
| Hurley (2010) | Creating and Sustaining Effective Partnerships to Advance Research on Youth With Serious Emotional and Behavioral Disorders |
| Jacelon (2010) | Scholar in Residence: an innovative application of the scholarship of engagement |
| Jacob (2000) | From sponsorship to partnership in academy-industry relations |
| Jenkins (2014) | Underpinning reflective practice in social care and housing provision through collaborative knowledge exchange |
| JesÃºs (2008) | Personnel participation as a key factor for success in maintenance program implementation: A case study |
| Jones (2020) | A micro-level view on knowledge co-creation through university-industry collaboration in a multi-national corporation |
| Jordan (2013) | Tackling alcohol misuse through screening and brief interventions: a knowledge transfer partnership: final report |
| Joubert (2015) | Academic Practitioner Partnerships: A Model for Collaborative Practice Research in Social Work |
| Kanani (2017) | AcademyHealth's Delivery System Science Fellowship: Training Embedded Researchers to Design, Implement, and Evaluate New Models of Care |
| Khan (2019) | An Analysis of Publication Productivity During Residency for 1506 Neurosurgical Residents and 117 Residency Departments in North America |
| Kislov (2017) | New Game, Old Rules? Mechanisms and Consequences of Legitimation in Boundary Spanning Activities |
| Kolympiris (2019) | Learning by seconding: Evidence from National Science Foundation rotators |
| Lalani (2018) | Building motivation to participate in a quality improvement collaborative in NHS hospital trusts in Southeast England: a qualitative participatory evaluation |
| Lang (2015) | Making research integral to home care services |
| Langeveld (2016) | Use of a knowledge broker to establish healthy public policies in a city district: a developmental evaluation |
| Latter (2009) | Implementing a clinical academic career pathway in nursing; criteria for success and challenges ahead |
| Lawn (2014) | Integration of primary health services: being put together does not mean they will work together |
| Leon (2011) | Integrating palliative care in public health: The Colombian experience following an International Pain Policy Fellowship |
| Levin (2007) | Building bridges in academic nursing and health care practice settings |
| Lewis (2011) | Being embedded: A way forward for ethnographic research |
| Lewis (2019) | Walking the Line: Brokering Humanitarian Identities in Conflict Research |
| Lightowler (2012) | Improving research use in the Third Sector: learning from work with APEX Scotland: project report |
| Lindamer (2009) | Establishing an implementation network: lessons learned from community-based participatory research |
| Lloyd (2017) | Collaborative action for person-centred coordinated care (P3C): an approach to support the development of a comprehensive system-wide solution to fragmented care |
| Losekoot (2008) | How change does not happen: The impact of culture on a submarine base |
| Lumsden (2018) | Public criminology, reflexivity and the enterprise university: Experiences of research, knowledge transfer work and co-option with police forces |
| Lundeen (2009) | Translating nursing knowledge into practice: An uncommon partnership |
| Lynette (2006) | Academic-practice partnerships in practice research: a cultural shift for health social workers |
| Manville (2019) | Supporting open innovation with the use of a balanced scorecard approach: a study on deep smarts and effective knowledge transfer to SMEs |
| Mariguddi (2022) | Co-researching OR researching with teachers in the space between: pitfalls and benefits |
| Martin (2008) | Innovation in the construction and property management industries: Case studies of the knowledge transfer partnership scheme |
| Massingham (2014) | The Researcher as Change Agent |
| Masterson (2020) | Redefining the role of the nurse academic in practice: A pilot study |
| Mayo-Gamble (2017) | Exploring Proxy Measures of Mutuality for Strategic Partnership Development: A Case Study |
| McAdam (2010) | The development of absorptive capacity-based innovation in a construction SME |
| McCabe (2015) | A model for collaborative working to facilitate knowledge mobilisation in public health |
| McCormick (2014) | A Regional Public Health Field Placement Program: Making an IMPACT |
| McEwen (2007) | In the know: Knowledge Transfer Partnerships - encouraging the development of an evidence-informed approach to practice through successful partnership between a University and a local authority  **Additional papers:**  *McEwen (2008) Promoting change through research and evidence-informed practice: A Knowledge Transfer Partnership project between a university and a local authority* |
| McLoughlin (2020) | Brokering knowledge into the public sector: understanding improvement facilitatorsâ€™ priorities in the redesign of hospital care |
| Meerhoff (2017) | Development and Evaluation of an Implementation Strategy for Collecting Data in a National Registry and the Use of Patient-Reported Outcome Measures in Physical Therapist Practices: Quality Improvement Study |
| Mickan (2020) | Embedded researchers in Australia: Survey of profile and experience across medical, nursing and midwifery and allied health disciplines |
| Mill (2014) | Qualitative Research in an International Research Program: Maintaining Momentum while Building Capacity in Nurses |
| Miller (2012) | Partnering to Translate Evidence-Based Programs to Community Settings: Bridging the Gap Between Research and Practice |
| Miller (2019) | Practice research enabler: enabling research in a social work practice context |
| Millward (2004) | Challenges in implementing design-led technologies in small manufacturing companies |
| Miszczak (2018) | The role of engaged scholarship and co-production to address urban challenges: a case study of the Cape Town Knowledge Transfer Programme |
| Mitchell (2009) | Partnerships for knowledge exchange in health services research, policy and practice |
| Moran (2019) | Mind the gap: is it time to invest in embedded researchers in regional, rural and remote health services to address health outcome discrepancies for those living in rural, remote and regional areas? |
| Morgan (2020) | Leveraging an Academic-Practice Partnership to Improve Maternal and Child Health Outcomes in North Carolina |
| Morris (2013) | Developing engagement, linkage and exchange between health services managers and researchers: Experience from the UK |
| Munkongsujarit (2014) | Researching Social Capital in R&D Management: A Case Study in High-Tech Industry |
| Murray (2020) | Evolution of the researcher-practitioner partner model and the role of academic research partners in Byrne Criminal Justice Innovation (BCJI) Grants |
| Newman (2011) | Partnership in knowledge creation: Lessons learned from a researcher-policy actor partnership to co-produce a rapid appraisal case study of South Australia's Social Inclusion Initiative |
| Newton (2017) | Evaluation Report: HEE North Central and East London & NIHR CLAHRC North Thames Clinical Nurse/Midwife/AHP (NMAHP) Academic Fellowship Scheme |
| Nielsen (2018) | Improving the Lives of Academia-Practitioner Boundary Spanners |
| Noble-Jones (2019) | The Asset-based Collaborative Working model: pragmatic action research in healthcare |
| O'Donoughue (2017) | The use of secondments as a tool to increase knowledge translation |
| Oancea (2021) | Research capacity-building in teacher education |
| Omidvar (2016) | R and D CONSORTIA AS BOUNDARY ORGANISATIONS: MISALIGNMENT AND ASYMMETRY OF BOUNDARY MANAGEMENT |
| Osborne (2020) | Red Dirt Thinking on first language and culturally responsive pedagogies in Anangu schools |
| Palmer (2019) | Perfecting the â€˜Elevator Pitchâ€™? Expert advice as locally-situated boundary work |
| Paradis (2017) | The Universityâ€“Public Health Partnership for Public Health Research Training in Quebec, Canada |
| Parke (2015) | Scholar-in-Residence: An Organizational Capacity-Building Model to Move Evidence to Action |
| Patel (2015) | Co-producing urban knowledge: experimenting with alternatives to 'best practice' for Cape Town, South Africa |
| Patterson (2014) | Walking with intangibles: experiencing organisational learning |
| Pattison (2021) | Florence Nightingale's legacy for clinical academics: A framework analysis of a clinical professorial network and a model for clinical academia |
| Paudel (2015) | Opioid availability and palliative care in Nepal: influence of an international pain policy fellowship |
| Penrod (2016) | Empowering Change Agents in Hierarchical Organizations: Participatory Action Research in Prisons |
| Perry (2015) | CAEP 2014 Academic Symposium: "How to make research succeed in your emergency department: How to develop and train career researchers in emergency medicine" |
| Phillips (2021) | Bugs in the Bed: Addressing the Contradictions of Embedded Science with Agile Implementation Research |
| Pitt (2017) | Pediatric Resident Academic Projects While on Global Health Electives: Ten Years of Experience at the University of Minnesota |
| Pomeroy (2003) | Dismantling some barriers to evidenced-based rehabilitation with 'hands-on' clinical research secondments: initial development |
| Probandari (2017) | Social multiplier effects: academics' and practitioners' perspective on the benefits of a tuberculosis operational research capacity-building program in Indonesia  **Additional papers:**  *Mahendradhata (2014) Embedding operational research into national disease control programme: lessons from 10 years of experience in Indonesia* |
| Pyett (2008) | Challenging our own practices in Indigenous health promotion and research |
| Reddy (2018) | Moving the needle towards health equity: A policy-driven transdisciplinary approach to address health disparities for vulnerable communities |
| Reupert (2011) | "A whole new language and a new world": Seconded teachers' experiences in a faculty of education |
| Richardson (2007) | Development of a critical care nursing research strategy: a tripartite approach |
| Riner (2015) | USING IMPLEMENTATION SCIENCE AS THE CORE OF THE DOCTOR OF NURSING PRACTICE INQUIRY PROJECT |
| Rivard (2010) | Promoting the use of measurement tools in practice: a mixed-methods study of the activities and experiences of physical therapist knowledge brokers |
| Robinson (2020) | Practice Transformation Driven through Academic Partnerships |
| Rose (2017) | The benefits of gaining research experience through a secondment |
| Rossi (2017) | Academic engagement as knowledge co-production and implications for impact: Evidence from Knowledge Transfer Partnerships |
| Rowley (2014) | Going beyond procedure: engaging with the ethical complexities of being an embedded researcher |
| Rycroft-Malone (2020) | Re-Framing the Knowledge to Action Challenge Through NIHR Knowledge Mobilisation Research Fellows Comment on "CIHR Health System Impact Fellows: Reflections on 'Driving Change' Within the Health System" |
| Sabri (2019) | Using collaborative research methodologies in humanitarian supply chains |
| Salminen-Karlsson (2008) | The interaction of academic and industrial supervisors in graduate education |
| Saltz (2017) | Predicting data science sociotechnical execution challenges by categorizing data science projects |
| Schreiber (2015) | Knowledge Translation and Implementation Special Series. The Use of a Knowledge Translation Program to Increase Use of Standardized Outcome Measures in an Outpatient Pediatric Physical Therapy Clinic: Administrative Case Report |
| Shaw (2018) | How can research mediators better mediate?: the importance of inward-looking processes |
| Shtasel (2015) | Developing a Pipeline for the Community-Based Primary Care Workforce and Its Leadership: The Kraft Center for Community Health Leadership's Fellowship and Practitioner Programs |
| Shurson (2021) | Utilizing Academicâ€“Service Partnerships to Advance the Care of Veterans |
| SimÃ³n (2018) | Workforce analytics: A case study of scholarâ€“practitioner collaboration |
| Sinfield (2012) | Placing interprofessional learning at the heart of improving practice: the activities and achievements of CLAHRC in Leicestershire, Northamptonshire and Rutland |
| Spoelstra (2019) | Building an Academic-Practice Partnership to Support Doctor of Nursing Practice Projects |
| Stark (2013) | Dementia knowledge transfer project in a rural area |
| Stefan (2014) | Success Factors and Obstacles in Academiaâ€“Industry Partnerships: A Case Study of a Graduate Program within the Bayerâ€“University of Cologne â€œPrivileged Partnershipâ€ |
| Stewart (2019) | Practical reflections on combining workshops and mentorships to build capacity in demand and use of evidence in government organisations  **Additional papers:**  *Jordaan (2018) Reflections on mentoring experiences for evidence-informed decision-making in South Africa and Malawi*  *Stewart (2018) Building capacity for evidence-informed decision making: an example from South Africa* |
| Steynor (2020) | Transdisciplinary co-production of climate services: a focus on process |
| Stoesz (2016) | A knowledge brokering process for challenging behaviours in special education |
| Thornley (2004) | Developing a research partnership: teachers as researchers and teacher educators |
| Tillyer (2014) | Researcherâ€“practitioner partnerships and crime analysis: a case study in action research |
| Townson (2016) | Outcomes from Applying Design-Led Innovation in an Australian Manufacturing Firm |
| Tran (2017) | Embedding research to improve program implementation in Latin America and the Caribbean |
| Trott (2009) | Core competencies for diversifying: Case study of a small business |
| Uneke (2018) | Promoting Researchers and Policy-Makers Collaboration in Evidence-Informed Policy-Making in Nigeria: Outcome of a Two-Way Secondment Model between University and Health Ministry |
| Upton (2013) | Phase 1 Evaluation of Lothianâ€™s Nursing, Midwifery and Allied Health Professions (NMAHP) Clinical Academic Research Careers (CARC) Scheme |
| VanderKooi (2018) | An Enhanced Actualized DNP Model to Improve DNP Project Placements, Rigor, and Completion |
| Varallyay (2020) | How does embedded implementation research work? Examining core features through qualitative case studies in Latin America and the Caribbean  **Additional papers:**  *Garcia-Cerde (2021) Embedded implementation research determinants in Latin American health systems*  *Varallyay (2020) Health system decision-makers at the helm of implementation research: development of a framework to evaluate the processes and effectiveness of embedded approaches* |
| Vigurs (2019) | Exorcising an Ethnography in Limbo |
| Vindrola-Padros (2019) | Addressing the challenges of knowledge co-production in quality improvement: learning from the implementation of the researcher-in-residence model |
| Walker (2008) | Collaborative academic/practitioner research in project management: Examples and Applications |
| Waqa (2013) | Knowledge brokering between researchers and policymakers in Fiji to develop policies to reduce obesity: a process evaluation |
| Ward (2012) | Exploring knowledge exchange: a useful framework for practice and policy |
| Ward (2021) | Embedding researchers into organisations: a study of the features of embedded research initiatives |
| Westlake (2020) | Direct observation in practice: co-developing an evidence-informed practice tool to assess social work communication  **Additional papers:**  *Barry (2017) The Islington 'Doing what counts: measuring what matters': evaluation report* |
| Westwood (2018) | Building clinical academic leadership capacity: sustainability through partnership |
| White (2019) | The soft side of knowledge transfer partnerships between universities and small to medium enterprises: an exploratory study to understand process improvement  **Additional papers:**  *White (2014) The implementation of an environmental management system in the not-for-profit sector* |
| Williamson (2019) | Increasing the capacity of policy agencies to use research findings: a stepped-wedge trial  **Additional papers:**  *Haynes (2018) The pivotal position of 'liaison people': Facilitating a research utilisation intervention in policy agencies* |
| Winkler (2019) | Do Researchers in Empirical Ethics Studies Have a Duty to Act Upon their Findings? Case Study in End-of-Life Decision Making |
| Wolfenden (2017) | Embedding researchers in health service organizations improves research translation and health service performance: the Australian Hunter New England Population Health example |
| Wormald (2009) | The integration of industrial design capability within UK SMEs: the challenges, opportunities and benefits |
| Wye (2019) | Knowledge brokers or relationship brokers? The rote of an embedded knowledge mobilisation team |
| Wye (2020) | Collective knowledge brokering: the model and impact of an embedded team |
| Wynn (2018) | Technology Transfer Projects in the UK: An Analysis of University - Industry Collaboration  **Additional papers:**  *Wynn (2017) Knowledge Transfer Partnerships and the entrepreneurial university*  *Wynn (2019) Context and entrepreneurship in Knowledge Transfer Partnerships with small business enterprises* |
| Yost (2014) | Tools to support evidence-informed public health decision making |
| Young (2018) | Researcher and policymaker dialogue: the Policy BUDDIES Project in Western Cape Province, South Africa |
| Yuan (2017) | Teacher identity development through action research: A Chinese experience |
| Zabel (2013) | Tackling alcohol misuse through screening and brief interventions in hospital trauma clinics: a knowledge transfer partnership |

## Appendix 6 – Distribution of studies by country


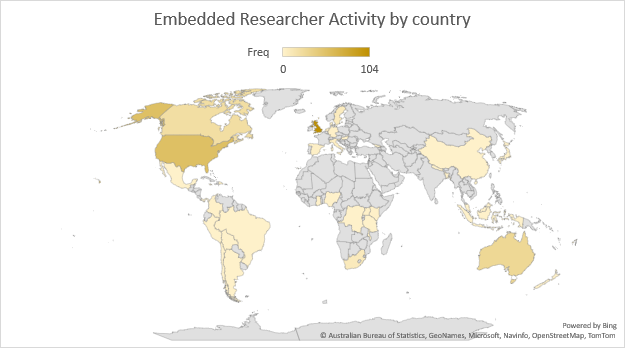


| Country | Number of studies | Country | Number of studies |
| --- | --- | --- | --- |
| England | 63 | Indonesia | 1 |
| Northern Ireland | 5 | Italy | 4 |
| Scotland | 12 | Japan | 2 |
| Wales | 9 | Jamaica | 2 |
| UK (unspecified) | 15 | Kenya | 2 |
| Europe-wide (unspecified) | 2 | Malawi | 1 |
| Argentina | 1 | Malaysia | 1 |
| Australia | 28 | Mexico | 1 |
| Bangladesh | 2 | Nepal | 1 |
| Barbados | 1 | Netherlands | 4 |
| Bolivia | 1 | New Zealand | 2 |
| Brazil | 1 | Nigeria | 1 |
| Canada | 21 | Panama | 1 |
| Chile | 1 | Peru | 1 |
| China | 1 | Saint Lucia | 1 |
| Colombia | 2 | Singapore | 1 |
| Denmark | 1 | Slovenia | 1 |
| Dominican Republic | 1 | South Africa | 8 |
| DR Congo | 1 | Spain | 3 |
| Fiji | 1 | Sweden | 7 |
| Georgia | 1 | Tanzania | 1 |
| Germany | 2 | Uganda | 2 |
| Ghana | 1 | USA | 53 |
| Hungary | 1 | Unclear | 1 |

## Appendix 7 – Full list of sectors represented

| **Discipline** | **Number of studies** |
| --- | --- |
| Clinical health; Primary or secondary care | 89 |
| Allied Health - Nutrition | 1 |
| Allied health - speech therapy | 2 |
| Allied health - physiotherapy | 5 |
| Allied health - various | 1 |
| Community mental health | 5 |
| Health service planning and integration | 10 |
| Public Health | 35 |
| Business/Management | 4 |
| Social care | 16 |
| Church/Religious Org | 1 |
| Construction - engineering/architectural/other services | 5 |
| Crime/law enforcement | 10 |
| Defence/military | 2 |
| Ecology/conservation | 2 |
| Education | 17 |
| Engineering | 3 |
| Government Depts - various | 4 |
| Housing | 2 |
| HR (Human Resources) | 1 |
| Industry - manufacturing | 16 |
| Industry - other | 12 |
| Industry (not specified) | 3 |
| International development | 3 |
| Management consultancy | 1 |
| Pharmaceutical Industry | 1 |
| Research Funder/Philanthropic Org | 2 |
| Tech company | 1 |
| Third sector - various | 7 |
| Tourism/ Cultural sector | 1 |
| Urban development | 2 |
| Various | 4 |
| Youth work | 2 |

## Appendix 8 – Evaluation designs implemented

### Evaluation design – structured methods

| Study design | Number of studies |
| --- | --- |
| RCT | 2 |
| Quasi-experimental | 6 |
| Interviews | 69 |
| Focus groups/workshops | 17 |
| Case study | 43 |
| Reflections | 43 |
| Description of processes | 13 |
| Select if described as process evaluation | 1 |
| Case series | 7 |
| (cross-sectional) survey | 39 |
| Diary and field notes | 25 |
| Secondary/routine data analysis | 5 |
| Ethnographic observation | 21 |
| Documentary analysis | 31 |
| Other | 19 |

### Evaluation design – using unstructured and reflective methods only

| Study design | Number of studies |
| --- | --- |
| Case study | 67 |
| Reflections | 69 |
| Description of processes | 13 |
| Case series | 10 |

## Appendix 9 – Codes and definitions for nature of embeddedness

Please see table below for codes and definitions for nature of embeddedness

| How was the researcher embedded? | |
| --- | --- |
| Physical co-location (confirmed) | *E.g. through details provided about share of time spent or resources provided to support physical co-location (e.g. a desk)* |
| Culturally/ Institutionally | *Look for examples where embedded researcher described as being embedded in cultural terms (i.e. a team member) or institutional terms (i.e. referred to as staff member of host org) – for example: References to being described as a team member in host team; References to being embedded in team culture; References to being set up to be staff member of host institution (e.g. through holding a joint contract or contract funded by host org – i.e. institutional barriers removed); References to embedded researcher being integral to larger programmes of work or aims of host org; References to embedded researcher being made to feel welcome/integrated etc.; Reference to researcher being viewed as fully participating in host org daily life; Reference to embedded researcher building things together with host team; References to policy people (in ‘reverse’ direction) being described as researchers in academic settings etc. ; References to transdisciplinarity* |
| Procedurally | *E.g. attends staff meetings or described as holding a particular function within the host team Look for examples where embedded researcher described specific procedures/processes that may be viewed as a form of embeddedness - for example: References to attending staff meetings; References to embedded researcher conducting specific tasks or occupying specific roles (e.g. tutor role or support role) within the host organization; References to embeddedness being sought through regular emails, calls etc. ; Also where study makes clear that embedded researcher was not a complete organizational member but did complete specific tasks* |
| Physical co-location (probable) | *E.g. where scheme is described but physical co-location inferred as optional* |
| Confirmed as embedded, nature not described | *Description vague – e.g. researcher described as embedding processes but unclear how* |
| Embeddedness sought but not achieved | *Where embeddedness was intended but was not achieved* |

## Appendix 10 – Terms used to describe embedded researcher interventions

Table 1: Glossary of terms overlapping with embedded researcher

| **Knowledge Transfer Partnership (KTP)** | **Knowledge Transfer Partnership** (KTP) generally describes a large programme of embedded research that has taken place over a number of years in the UK that has sought to engage universities with commercial, policy and practice organisations for the benefit of both (it was previously known as the teaching company scheme). A KTP project involves a knowledge base (an academic partner) and a business or organisation together on a project (UKRI 2022). The academic partner employs a graduate (a Knowledge Transfer Associate) who is based within a non-academic organisation and co-managed between the academic and non-academic partner. The nature of the project involves developing new products, services or way of working through academic input, and in creating the map studies were included where there was discussion of research or evaluation in the creation of the project.  ***Relevance to Embedded Researchers:*** There is substantial, although not complete, overlap between the function of an embedded researcher and a KTP associate. Many studies described a KTP project as opening the door for further collaboration on research-related activities. However, not all evaluations of KTP were included, with studies that did not describe incorporating processes for generating a more research active culture being excluded. While all KTP require academic involvement, the nature of this involvement does not appear in all cases to involve incorporating academic research methods into the collaboration.  **The map includes 24 studies** that evaluate a KTP and use this term to signify the embedded researcher intervention.  **Schemes from other countries have not been included here e.g. Young Researchers in the Economy (Slovenia)** |
| --- | --- |
| **Knowledge Broker (KB)** | **Knowledge Brokers (KBs)** are ‘persons or organisations that facilitate the creation, sharing, and use of knowledge’ as well as persons occupying roles to ‘maintain links between researchers and their audiences via the appropriate translation of research findings’ (Meyer 2010, p119). KBs occupy a ‘bridging’ role linking communities of evidence users and generators of evidence (Dobbins et al. 2009), with knowledge brokering described both in relational and transformational terms (Meyer 2010, Robeson et al. 2008).  ***Relevance to Embedded Researchers:*** Embedded Researchers are likely to undertake knowledge brokering activities, particularly where these involve building relationships and transforming knowledge for local use, although may also produce research or facilitate the production of research.  **The map includes 15 studies** that use this term to signify the embedded researcher intervention. |
| **Researchers-in-Residence (RiR)** | **Researchers-in-Residence (RiR)** are Experienced researchers (usually academics) embedded individually or as a team into a service frontline or management team for a substantial period of time – several months or years (Marshall et al. 2022, Marshall et al. 2016). The researcher is a ‘staff member’ of the service team with ‘daily interaction’ and ‘immersion’, whilst maintaining their affiliation with their university (Vindrola-Padros et al. 2019).The role usually involves physical co-location (Marshall et al, 2022), although this was not explicit in early definitions (e.g. (Marshall et al. 2016)). They integrate and negotiate their academic expertise with colleagues’ practice-based expertise (Marshall et al. 2016) to co-produce and mobilise “actionable knowledge in context” (Marshall et al. 2022) and increase the service’s research capacity (Vindrola-Padros et al. 2019).  ***Relevance to Embedded Researchers:*** Researchers-in-Residence are viewed as a subset of Embedded Researchers here, but are described elsewhere as synonymous with ‘embedded researchers’ (Marshall et al. 2022, Marshall et al. 2016, Vindrola-Padros et al. 2019). Our definition of Embedded Researchers is broader by including non-service sectors (e.g. manufacturing industry and policy organisations); practitioners and service managers embedded into research teams; and shorter placements (for at least one month). Additionally, an Embedded Researcher may not be physically co-located or a staff member of the host organisation.  **The map includes 12 studies** that use this term to signify the embedded researcher intervention. See also scholar-in-residence. |
| **Practitioner researcher** | **Practitioner researchers** are researchers working within practice settings and with a mainly practitioner background carrying out research in situ and studying their own or their organisation’s practice. The emergence of practitioner-researchers reflects a more knowledge-based economy with a workforce that is highly trained, including in terms of research methods training, that has the skills to draw knowledge from its own practice and where practice is increasingly viewed as a site for learning (Jarvis 2000). Practitioner research has been described by others as a form of action research within one’s own practice, and is tied to ambitions around personal growth and practice improvement (Ragland 2006). There are also parallels with other approaches described above, where traditional distinctions and hierarchies in research production are weakened through practitioner research including a recognition that practitioners are best places to learn systematically about their own practice (Jarvis 2000).  ***Relevance to Embedded Researchers:*** Practitioner research takes several different forms, although may follow some of the same steps as other practices described above including action research. There are parallels and overlaps with embedded researchers, particularly around an ambition to develop a more research active culture through generating practice-based research. However, embedded researcher interventions require an element of dual affiliation where practitioner research does not, although dual affiliation may be present to support practitioner research. We also note that practitioner researchers are distinct from practitioner-research partnerships, which involve intermural collaboration (Rojek et al. 2012).  **The map includes 8 studies** that use ‘practitioner researcher’ or an allied term such as ‘clinician researcher’, ‘teacher researcher’ or ‘nurse researcher’ to signify the embedded researcher intervention.  **Other studies in the map use terms such as ‘clinical academic’ and ‘clinician scientist’ to signify embedded researchers who hold joint academic and clinical posts (‘pracademics’). The term ‘pracademic’ is defined separately in our full Glossary.** |
| **Academic-service partnerships** | **Academic-service partnerships** are organisational level interventions that involve the development of strategic relationships between academic and service institutions that are intended to deliver mutual benefits (Sadeghnezhad et al. 2018).  ***Relevance to Embedded Researchers:*** Academic-service partnerships may incorporate embedded researchers as a way of enhancing research activity within service organisations, although this is not a fundamental principle of academic-service partnerships.  **The map includes 8 studies** that use this term or allied terms such as ‘academic-clinical partnership’ and ‘academic-practice partnership’ to signify the embedded researcher intervention. |
| **Knowledge translation (KT)** | **Knowledge Translation (KT)** (sometimes used interchangeably with **Knowledge Mobilisation** and **Knowledge-to-Action**) is often used as a broad term for activities supporting the use of **research**-based knowledge in policy, practice and services (CIHR 2015), also called Research Utilisation and Uptake. Other forms of knowledge (such as practice-derived and experiential knowledge) may also be within scope (Rushmer et al. 2019). Knowledge Translation activities can be carried out solely by knowledge producers (‘push’ – for example evidence summaries as one-way **‘knowledge transfer’**); solely by knowledge users (‘pull’ – for example, **‘knowledge champions’** within services); or involve two-way dialogue, co-creation and mutual learning (**‘knowledge exchange’**) (Nguyen et al. 2020, Rushmer et al. 2019). Varied conceptualisations of Knowledge Translation may require dynamic interactions between knowledge producers and users (CIHR 2015); include knowledge synthesis (CIHR 2015); or exclude “the simple dissemination of knowledge” by researchers (Straus et al. 2009). In the clinical context, the term ‘knowledge translation’ is also used (along with **“bench to bedside”** and **“translational medicine”**) to describe the use of laboratory evidence to create treatments, diagnostic tests and other interventions; and the diffusion and implementation of these technologies (**‘technology transfer’**) at scale in clinical practice (Greenhalgh and Wieringa 2011, Pearson et al. 2012).  ***Relevance to Embedded Researchers:*** Embedded Researchers are complex two-way knowledge translation **(‘exchange’)** interventions:– “disruptors of organisational cultures” who have a dual affiliation to the worlds of research producers and research users, and build a culture of evidence use in a policy or practice organization (Kneale et al. 2021). An Embedded Researcher may undertake discrete knowledge translation activities; integrate research evidence with other forms of knowledge; and facilitate evidence use via building relationships and being a team member in their host organisation. The Researcher may directly apply evidence to organisational and practitioner decision-making during their placement (instrumental use) as well as indirectly influencing decisions in the longer-term via an ‘enlightenment’ effect on the understandings of policymakers and practitioners (Kneale et al. 2021).  **The map includes 7 studies** that use ‘translation’ (research translation), or an allied term such as ‘knowledge transfer’, 'knowledge mobilisation' or 'knowledge exchange' to signify the embedded researcher intervention. |
| **Action Research and Action Researcher** | **Action Research (and Action Researcher)** involves incorporating collective and individual change alongside enhanced understanding, in response to a practical problem (Altrichter et al. 2002). The classic action research process comprises evolving cycles of planning, action, data collection/ analysis (‘observation’) and critical reflection (Altrichter et al. 2002), in which change and understanding are interdependent (Bradbury-Huang 2010), and mutual learning emerges.  ***Relevance to Embedded Researchers:*** An Embedded Researcher can facilitate Action Research within their host organisation to build research capacity in addition to achieving practice or organisational change. However, in many cases of organisational action research, either a research facilitator acts as a consultant (fully external to the organisation) and would not be defined as an Embedded Researcher; or at the other end of the spectrum, practitioner-researchers research their own practice or organisation without the involvement of a separate research organisation. Where a university provides technical support for practitioner action research, or where a practitioner carries out an “insider action research doctorate” (Coghlan 2007), there may be a blurred boundary that meets our definition of Embedded Researcher.  **The map includes 5 studies** that use ‘action research’ or ‘action researcher’.to signify the embedded researcher intervention. |
| **Participatory Action Research** (PAR) | **Participatory Action Research** (PAR) shares similarities with action research in that it involves collective and self-reflective inquiry that researchers and participants undertake (Baum et al. 2006). PAR approaches have their routes in a social justice agenda and seek to disrupt traditional power disparities that are characteristic of traditional public health research (Baum 2016). PAR is more frequently applied to community-based research than is the case with action research (above), which is often used in the context of organisational change (Vaughn and Jacquez 2020).  ***Relevance to Embedded Researchers:*** In many ways the goals of PAR are shared with Embedded Researcher interventions where there is an aim to create a more research active culture through blurring the lines between the ‘researched and researchers’ (Baum et al. 2006); we can view Embedded Researchers as seeking to undertake a similar endeavour through enabling research to be more deeply integrated into everyday practice. However, PAR has its routes in a social justice and community engagement; in the context of an embedded researcher intervention taking place within an organisation, the type of power differential that PAR seeks to disrupt may not be as obvious.  **The map includes 4 studies** that use this term to signify the embedded researcher intervention, **and a further 4 studies** that instead use the term ‘participatory research’ which describes a broader family of methods (see Vaughn and Jacquez (2020)). |
| **Industrial doctoral programme (Industrial PhD)** | An **Industrial PhD student researcher** is employed by an industrial organisation during their doctorate (Nielsen et al. 2018), which may be part-time alongside their usual type of work. They are jointly supervised by their employer (the industrial supervisor) and an academic supervisor (Salminen-Karlsson and Wallgren 2008). The doctoral studies focus on the practical needs of the business as well as being original academic enquiry.  ***Relevance to Embedded Researchers:*** Industrial doctoral students are generally a subset of Embedded Researchers because the student researcher is integrated into and often co-located with the employing organisation, and the research focus is co-created by the student, university and industry (Nielsen et al. 2018). This is in contrast to part-time doctoral students who carry out their research in the setting in which they are employed but where there is not joint influence by and two-way benefit for their employer and the university - these students are not defined as Embedded Researchers.  **The map includes 4 studies** that use **the terms ‘business PhD, ‘joint postgraduate programme’ or ‘collaborative doctoral training’ to signify this type of embedded researcher intervention.**  Other studies also evaluate other types of doctoral students as embedded researchers. |
| **Scholar-in-residence** | **Scholar-in-residence** has many similarities with ‘professor-in-residence’  and involves co-location of an academic member of staff in a policy or practice setting to promote research activity through a blending of two organisational cultures (Jacelon et al. 2010). Scholar-in-residence is also used to describe international cooperation between two academic organisations, although this second usage is not of interest here  ***Relevance to Embedded Researchers:*** There appears to be substantial conceptual overlap between our understanding of the definition of an ‘embedded researcher’ and a scholar-in-residence. However, our definition of the term embedded researchers appears to be more flexible in terms of the ‘direction’ of embeddedness (allowing for researchers from policy settings to be embedded into practice settings); as a term ‘embedded researcher’ may also help to blur identities compared to ‘scholar’-in- residence, which maintains the academic-policy/practice distinction.  **T**he map includes **3 studies that use this term to signify the embedded researcher intervention.** |

Additionally, the following generic terms were each used in at least 3 studies in our map to signify an intervention or job role which we have included as an embedded researcher intervention: Fellow/Fellowship; secondment/secondee; internship or placement; mentor (and allied terms - coach, researcher buddy); academic supervisor (and allied terms - academic mentor, academic advisor, clinical supervisor); doctoral student (and allied terms - student placement and student trainees); change agent; facilitator (and allied terms such as 'research co-ordinator'); and evaluator (and terms which included ‘evaluation’).

## Appendix 11

Table 2: Latent Class Analysis - Model fit statistics for different numbers of classes

| **Number of classes** | **N** | **Log likelihood** | **Df** | **AIC** | **BIC** | **Entropy** |
| --- | --- | --- | --- | --- | --- | --- |
| **3** | 229 | -1284.898 | 35 | 2639.795 | 2759.976 | 0.999 |
| **4** | 229 | -1254.753 | 47 | 2603.507 | 2764.892 | 0. 999 |
| **5** | 229 | -1226.56 | 59 | 2571.12 | 2773.709 | 0.971 |
| **6** | 229 | -1218.568 | 70 | 2577.135 | 2817.496 | 0.970 |

# References

Altrichter H, Kemmis S, McTaggart R, Zuber‐Skerritt O (2002) The concept of action research. *The learning organization*.

Baum F, MacDougall C, Smith D (2006) Participatory action research. *Journal of epidemiology and community health* 60**:** 854.

Baum FE (2016) Power and glory: applying participatory action research in public health. SciELO Public Health.

Bradbury-Huang H (2010) What is good action research? Why the resurgent interest? *Action Research* 8**:** 93-109.

CIHR (2015) *A guide to knowledge translation at CIHR: integrated and end of grant approaches.* Canadian Institutes of Health Research. <http://www.cihr-irsc.gc.ca/e/45321.html>

Coghlan D (2007) Insider action research doctorates: Generating actionable knowledge. *Higher Education* 54**:** 293-306.

Dobbins M, Robeson P, Ciliska D, Hanna S, Cameron R, O'Mara L, DeCorby K, Mercer S (2009) A description of a knowledge broker role implemented as part of a randomized controlled trial evaluating three knowledge translation strategies. *Implementation Science* 4**:** 1-9.

Greenhalgh T, Wieringa S (2011) Is it time to drop the ‘knowledge translation’metaphor? A critical literature review. *Journal of the Royal Society of Medicine* 104**:** 501-509.

Jacelon CS, Donoghue LC, Breslin E (2010) Scholar in residence: an innovative application of the scholarship of engagement. *Journal of Professional Nursing* 26**:** 61-66.

Jarvis P (2000) The practitioner–researcher in nursing. *Nurse Education Today* 20**:** 30-35.

Kneale D, Lester S, Stansfield C, Thomas J (2021) What are ‘Embedded Researchers’ and what influence could they have?

Marshall M, Eyre L, Lalani M, Khan S, Mann S, de Silva D, Shapiro J (2016) Increasing the impact of health services research on service improvement: the researcher-in-residence model. *Journal of the Royal Society of Medicine* 109**:** 220-225.

Marshall M, Davies H, Ward V, Waring J, Fulop NJ, Mear L, O’Brien B, Parnell R, Kirk K, Reid B (2022) Optimising the impact of health services research on the organisation and delivery of health services: a mixed-methods study. *Health and Social Care Delivery Research* 10**:** 1-182.

Meyer M (2010) The rise of the knowledge broker. *Science communication* 32**:** 118-127.

Molleman G, Fransen G (2012) Academic collaborative centres for health promotion in the Netherlands: building bridges between research, policy and practice. *Family practice* 29**:** i157-i162.

Nguyen T, Graham ID, Mrklas KJ, Bowen S, Cargo M, Estabrooks CA, Kothari A, Lavis J, Macaulay AC, MacLeod M (2020) How does integrated knowledge translation (IKT) compare to other collaborative research approaches to generating and translating knowledge? Learning from experts in the field. *Health Research Policy and Systems* 18**:** 1-20.

Nielsen RK, Poulfelt F, Buono A (2018) Improving the lives of academia-practitioner boundary spanners. *Academy of Management Proceedings.* Academy of Management.

Pearson A, Jordan Z, Munn Z (2012) Translational science and evidence-based healthcare: a clarification and reconceptualization of how knowledge is generated and used in healthcare. *Nursing research and practice* 2012.

Ragland BB (2006) Positioning the practitioner-researcher: Five ways of looking at practice. *Action Research* 4**:** 165-182.

Robeson P, Dobbins M, DeCorby K (2008) Life as a knowledge broker in public health. *Journal of the Canadian Health Libraries Association/Journal de l'Association des bibliothèques de la santé du Canada* 29**:** 79-82.

Rojek J, Smith HP, Alpert GP (2012) The prevalence and characteristics of police practitioner–researcher partnerships. *Police Quarterly* 15**:** 241-261.

Rushmer R, Ward V, Nguyen T, Kuchenmüller T (2019) Knowledge translation: key concepts, terms and activities. In: *Population health monitoring.* Springer, pages 127-150.

Rycroft-Malone J, Wilkinson JE, Burton CR, Andrews G, Ariss S, Baker R, Dopson S, Graham I, Harvey G, Martin G (2011) Implementing health research through academic and clinical partnerships: a realistic evaluation of the Collaborations for Leadership in Applied Health Research and Care (CLAHRC). *Implementation Science* 6**:** 1-12.

Sadeghnezhad M, Nabavi FH, Najafi F, Kareshki H, Esmaily H (2018) Mutual benefits in academic-service partnership: An integrative review. *Nurse Education Today* 68**:** 78-85.

Salminen-Karlsson M, Wallgren L (2008) The interaction of academic and industrial supervisors in graduate education. *Higher Education* 56**:** 77-93.

Straus SE, Tetroe J, Graham I (2009) Defining knowledge translation. *CMAJ* 181**:** 165-168.

UKRI (2022) *Knowledge Transfer Partnership guidance*. <https://www.ukri.org/councils/innovate-uk/guidance-for-applicants/guidance-for-specific-funds/knowledge-transfer-partnership-guidance/> (accessed November 27th 2022).

Vaughn LM, Jacquez F (2020) Participatory research methods–Choice points in the research process. *Journal of Participatory Research Methods* 1**:** 13244.

Vindrola-Padros C, Eyre L, Baxter H, Cramer H, George B, Wye L, Fulop NJ, Utley M, Phillips N, Brindle P (2019) Addressing the challenges of knowledge co-production in quality improvement: learning from the implementation of the researcher-in-residence model. *BMJ quality & safety* 28**:** 67-73.
